# Tracking human population structure through time from whole genome sequences

**DOI:** 10.1371/journal.pgen.1008552

**Published:** 2020-03-09

**Authors:** Ke Wang, Iain Mathieson, Jared O’Connell, Stephan Schiffels

**Affiliations:** 1 Department of Archaeogenetics, Max Planck Institute for the Science of Human History, Jena, Germany; 2 Department of Genetics, Perelman School of Medicine, University of Pennsylvania, Philadelphia, Pennsylvania, United States of America; 3 23andMe Inc., Mountain View, California, United States of America; Aarhus University, DENMARK

## Abstract

The genetic diversity of humans, like many species, has been shaped by a complex pattern of population separations followed by isolation and subsequent admixture. This pattern, reaching at least as far back as the appearance of our species in the paleontological record, has left its traces in our genomes. Reconstructing a population’s history from these traces is a challenging problem. Here we present a novel approach based on the Multiple Sequentially Markovian Coalescent (MSMC) to analyze the separation history between populations. Our approach, called MSMC-IM, uses an improved implementation of the MSMC (MSMC2) to estimate coalescence rates within and across pairs of populations, and then fits a continuous Isolation-Migration model to these rates to obtain a time-dependent estimate of gene flow. We show, using simulations, that our method can identify complex demographic scenarios involving post-split admixture or archaic introgression. We apply MSMC-IM to whole genome sequences from 15 worldwide populations, tracking the process of human genetic diversification. We detect traces of extremely deep ancestry between some African populations, with around 1% of ancestry dating to divergences older than a million years ago.

## Author summary

Human demographic history is reflected in specific patterns of shared mutations between the genomes from different populations. Here we aim to unravel this pattern to infer population structure through time with a new approach, called MSMC-IM. Based on estimates of coalescence rates within and across populations, MSMC-IM fits a time-dependent migration model to the pairwise rate of coalescences. We implemented this approach as an extension to existing software (MSMC2), and tested it with simulations exhibiting different histories of admixture and gene flow. We then applied it to the genomes from 15 worldwide populations to reveal their pairwise separation history ranging from a few thousand up to several million years ago. Among other results, we find evidence for remarkably deep population structure in some African population pairs, suggesting that deep ancestry dating to one million years ago and older is still present in human populations in small amounts today.

## Introduction

Genomes harbor rich information about population history, encoded in patterns of mutations and recombinations. Extracting that information is challenging, since in principle it requires reconstructing thousands of gene genealogies separated by ancestral recombination events, using only the observable pattern of shared and private mutations along multiple sequences. One important innovation was the Sequentially Markovian Coalescent (SMC) model [1,2], which is an approximate form of the ancestral recombination graph that can be fitted as a Hidden Markov model along the sequence. This approach has been used to infer demographic history in methods like PSMC [3], MSMC [4], diCal [5,6] and SMC++ [7].

These methods estimate one or both of two important aspects of population history: i) The history of the effective population size, and ii) the history of population structure. The second aspect, which entails reconstructing the timing and dynamics of population separation requires a non-trivial choice of parameterization: While methods like diCal2 [5], as well as many methods based on the joint site frequency spectrum [8–11] use an explicit population model with split times, migration rates or admixture events, MSMC [4] introduced the concept of the relative cross coalescence rate to capture population separations in a continuously parameterized fashion. The main advantage of that approach is that it does not require the specification of an explicit model, but can be applied hypothesis-free to estimate key aspects of population separation, for example the time at which lineages are half as likely to coalesce between rather than within populations, which is often used as a heuristic estimate for the divergence time between the populations. A disadvantage is that other important aspects of population separation, like post-split or archaic admixture, are non-trivially encoded in features of the cross-coalescence rate other than this mid-point. As a consequence, it is difficult to interpret the cross-coalescence rate in terms of actual historical events.

Here, we propose an approach to overcome the disadvantages of the relative cross coalescence rate, while maintaining the continuous character of population separation from MSMC without explicitly specifying a complex population phylogeny. We present a new method MSMC-IM, which fits a continuous Isolation-Migration (IM) model to the distribution of coalescence times, estimated from MSMC’s piecewise constant model. In MSMC-IM, separation and migration between a pair of populations is quantified by a piecewise constant migration rate across populations, and piecewise constant population size changes within each population. We apply our method on world-wide human genomic data from the Simons Genome Diversity Project (SGDP) [12] to investigate the history of global human population structure.

## Results

### Estimating pairwise coalescence rates with MSMC2 and fitting an IM model

**Fig 1:**
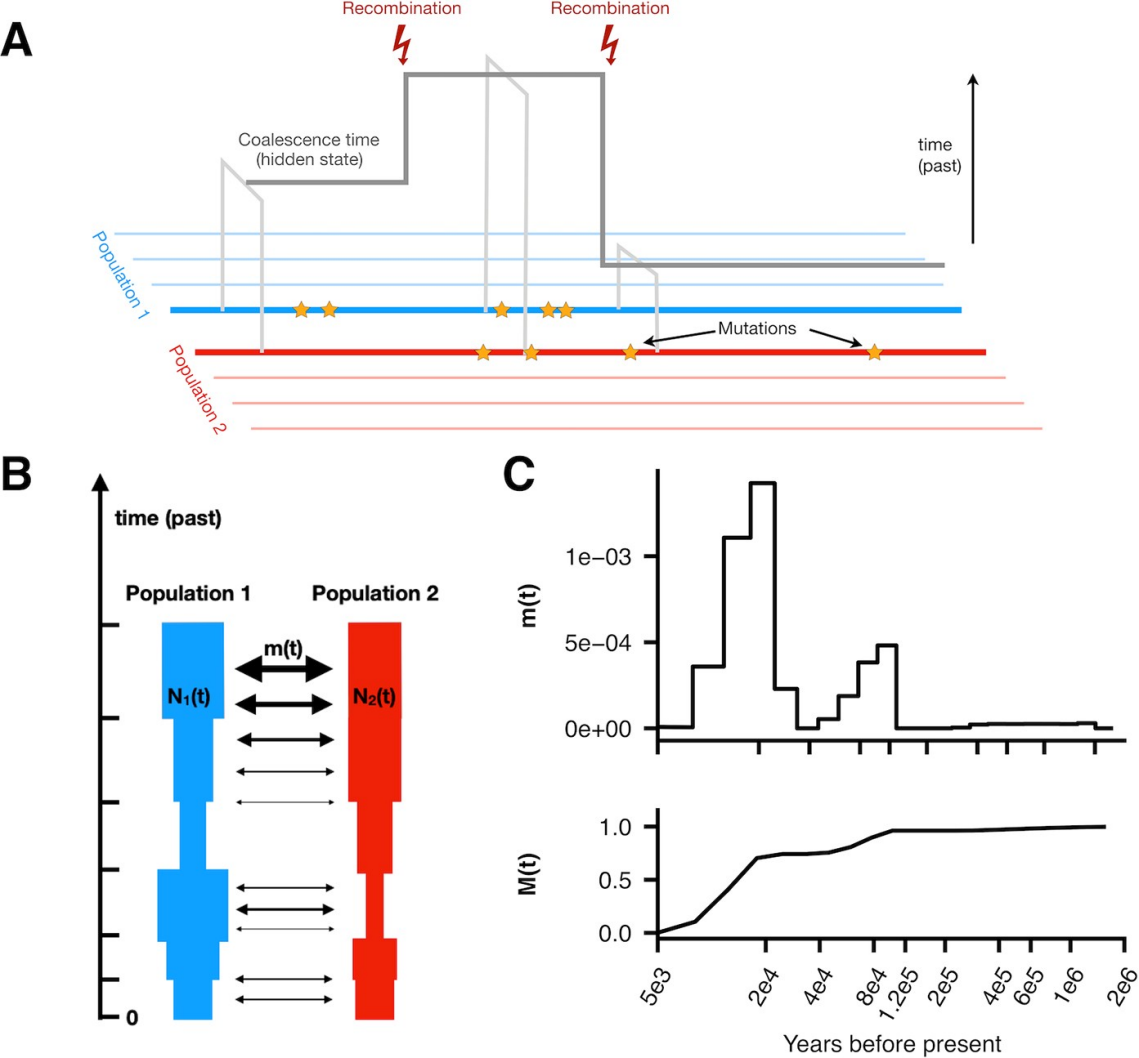
Schematic of MSMC2 and MSMC-IM. (A) MSMC2 analyses patterns of mutations between pairs of haplotypes to estimate local coalescence times along the genome. (B) MSMC-IM fits an isolation-migration model to the pairwise coalescence rate estimates, with time-dependent population sizes and migration rate. (C) As a result, we obtain the migration rate over time, *m(t)*, and the cumulative migration probability, *M(t)*, which denotes the probability for lineages to have merged by the time *t* and which we use to estimate fractions of ancestry contributed by lineages diverged deeper than time *t*.

To model the ancestral relationship between a pair of populations, we developed an isolation-migration model with a time-dependent migration rate between a pair of populations, which we call MSMC-IM. The approach requires time-dependent estimates of pairwise coalescence rates within and across two populations. To estimate these rates, we use an extension of MSMC [4], called MSMC2, which was first introduced in Malaspinas et al. 2016 [13] (Fig 1A, Methods). MSMC2 offers two key advantages over MSMC [4]. First, the pairwise coalescence model in MSMC2 is exact within the SMC’ framework [2], whereas MSMC’s model uses approximations that cause biases in rate estimates for larger number of haplotypes (S1 Fig). Second, since MSMC2 uses the pairwise tMRCA distribution instead of the first tMRCA distribution, it estimates coalescence rates within the entire range of coalescence events between multiple haplotypes, which ultimately increases resolution not just in recent times but also in the deep past. These two improvements are crucial for our new method MSMC-IM, which relies on unbiased coalescence rate estimates within and across populations, in particular in the deep past. Specifically, MSMC2 recovers simulated population size histories (with human-like parameters) well up to 3 million years ago, while keeping the same high resolution in recent times as MSMC (S1 Fig).

Given MSMC2’s estimates of time-dependent coalescence rates within populations, *λ*_*11*_*(t)* and *λ*_*22*_*(t)*, and across populations, *λ*_*12*_*(t)*, we use MSMC-IM to fit an Isolation-Migration (IM) model to those three coalescence rates (see Methods). MSMC-IM’s model assumes two populations, each with its own population size *N*_*1*_*(t)* and *N*_*2*_*(t)*, and a piecewise-constant symmetric migration rate *m(t)* between the two populations (Fig 1B, see Methods and S1 Text for details). Expressing the separation history between two populations in terms of a variable migration rate instead of the more heuristic relative cross coalescence rate facilitates interpretation, while maintaining the freedom to analyze data without having to specify an explicit model of splits and subsequent gene flow. Of the new parameters, the time-dependent migration rate *m(t)* is arguably the most interesting one, and it can be visualized in two ways (Fig 1C). First, the rates themselves through time visualize the timing and dynamics of separation processes, and second, the cumulative migration probability *M(t)* defined as

$$M(t)=1-exp\left(-\int_{0}^{t}m(t\prime )dt\prime \right)$$

which can be understood as the proportion of ancestry that has already merged at time *t*, and which makes it possible to quantify proportions of gene flow or archaic ancestry through time, as illustrated below. Being by definition monotonically increasing and bounded between 0 and 1, *M(t)* also turns out to be numerically close to the relative cross coalescence rate from MSMC [4]. When *M(t)* becomes very close to 1, it means that lineages between the two extant populations have completely mixed into essentially one population. As a technical caveat, this means that at that time point our three-parameter model is overspecified. To avoid overfitting, we therefore employ regularization on *m(t)* and the difference of the two population sizes (see Methods).

### Evaluating MSMC-IM with simulated data

**Fig 2:**
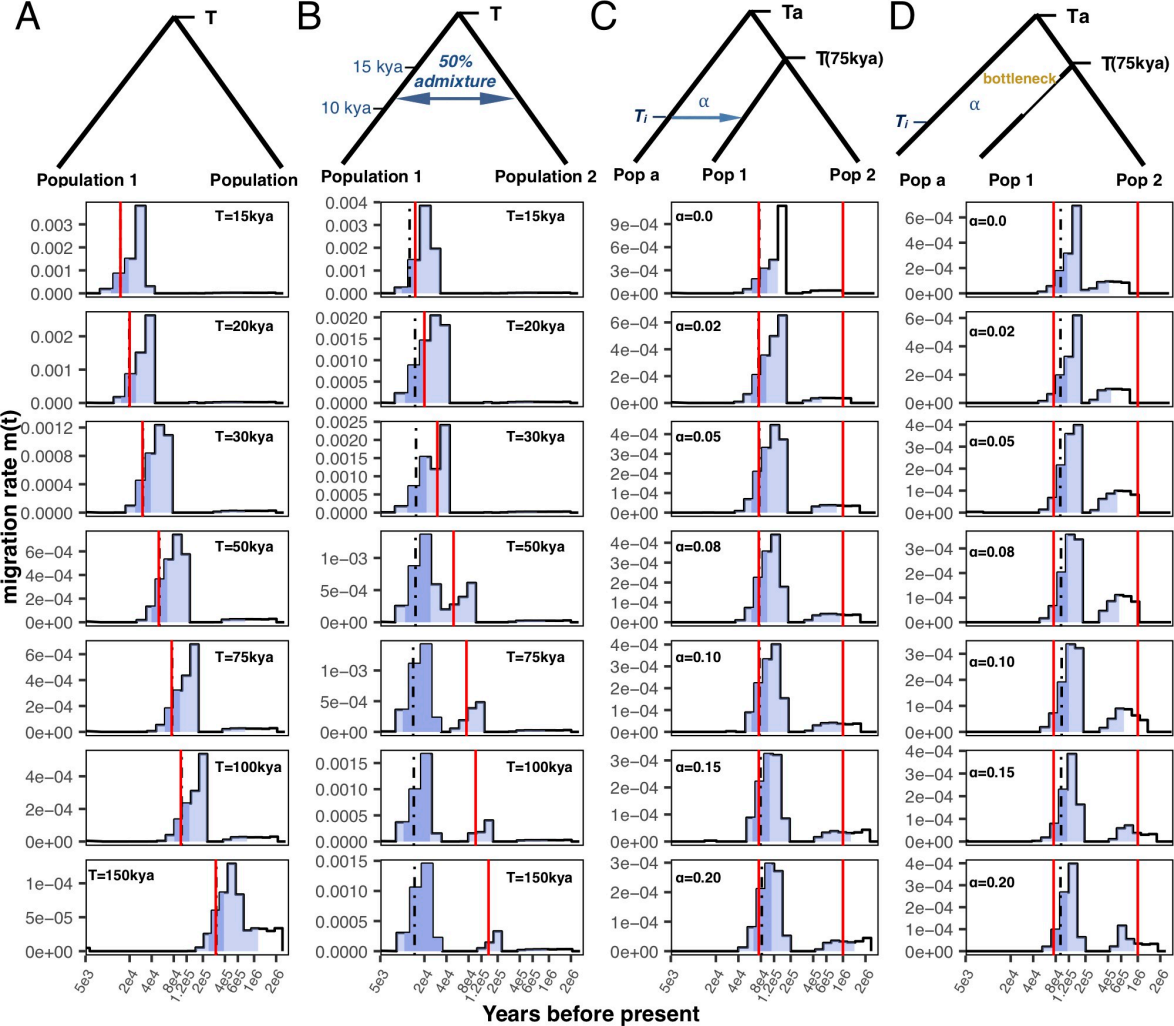
Simulation results. (A) *Clean-split* scenario: Two populations with constant size 20,000 each diverged at split time *T* in the past, varying from 15kya to 150kya. (B) *Split-with-migration* scenario. Similar to A), with *T* varying between 15-150kya, and a post-split time period of symmetric migration (amounting to a total migration rate of 0.5 in both directions) between 10 and 15kya. (C) *Split-with-archaic-admixture* scenario: Similar to A), with *T* = 75kya, and population 1 receiving an admixture pulse at 30kya from an unsampled population that separates from the ancestral population at 1 million years ago. The admixture rate varies from 0% to 20%. (D) *Split-with-archaic-admixture&bottleneck* scenario: Similar to C), but with an added population bottleneck with factor 30 in population 1 between 40-60kya. Solid red lines indicate split times in all panels. In all plots, the blue light blue shading indicates the interval between 1–99% of the cumulative migration probability, the dark blue shading from 25–75%, and the black dashed vertical line indicates the median.

We illustrate MSMC-IM by applying it to several series of simulated scenarios of population separation (see Methods). First, the *clean-split* scenario consists of an ancestral population that splits into two subpopulations at time T (Fig 2A). Second, the *split-with-migration* scenario adds an additional phase of bidirectional gene flow between the populations after they have split (Fig 2B). Third, the *split-with-archaic-admixture* scenario involves no post-split gene flow, but contains additional admixture into one of the two extant populations from an unsampled “ghost” population, which splits from the ancestral population (Fig 2C) at time *T*_*a*_*>T*. In addition, to understand how MSMC-IM behaves under asymmetric demographic histories in the two populations, we consider the *archaic-admixture-with-bottleneck*-scenario (see Fig 2D). For each scenario, we simulated 8 haplotypes (four from each population), used human-like evolutionary parameters and varied one key parameter to create a series of related scenarios (see Methods). As discussed further below, to test internal consistency, we confirmed that MSMC-IM is able to infer back its own model, using simulations based on some of the genomic inferences carried out below.

In the *clean-split* scenario, we find that MSMC-IM’s inferred migration rate *m(t)* displays a single pulse of migration around the simulated split time *T* (Fig 2A). This is expected, since in our parametrization, a population split corresponds to an instantaneous migration of lineages into one population at time *T*, thereby resulting in a single pulse of migration. In the *split-with-migration* series, we expect two instead of one pulse of migration: one at time *T*, as above, and a second more recent one around the time of post-split migration. In cases where the split time and migration phase are separated by more than around 20,000 years, this is indeed what we see (Fig 2B), although with some noise around this basic pattern. For less time of separation of the two migration pulses, MSMC-IM is not able to separate them in this scenario.

**Fig 3:**
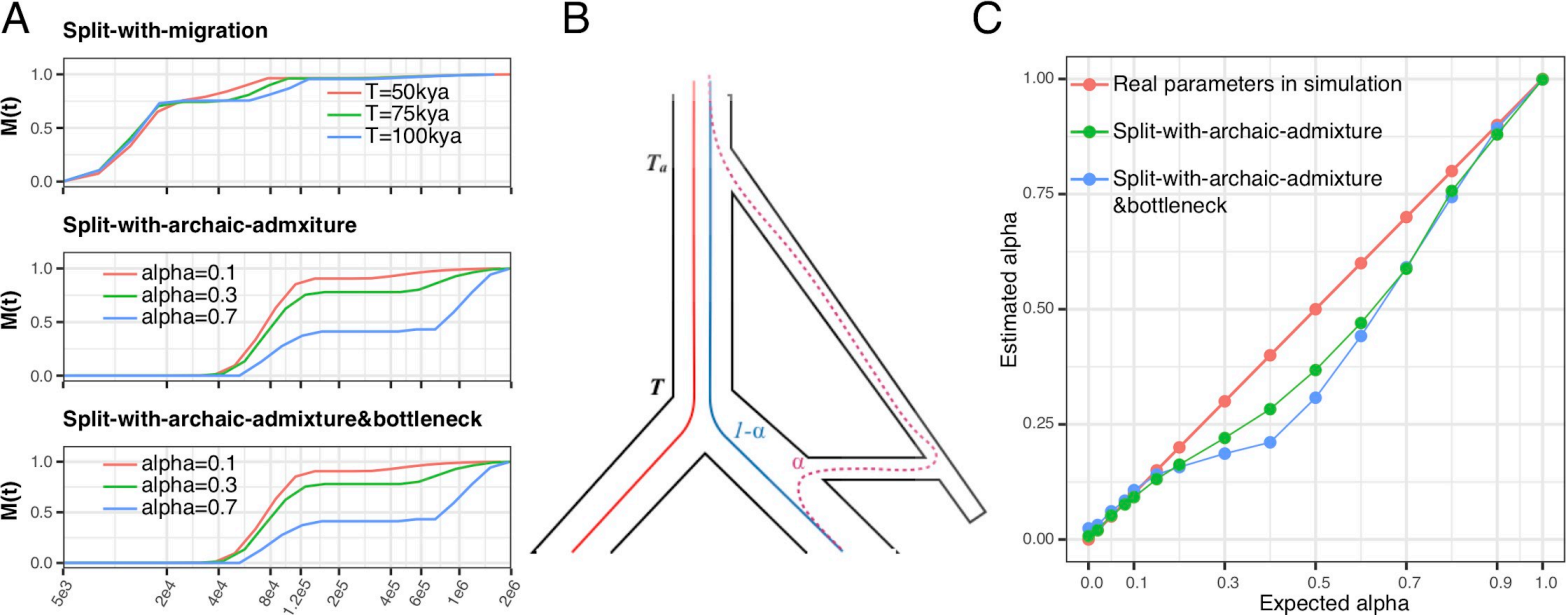
Evaluating admixture proportions through *M(t)*. (A) The cumulative migration probability *M(t)* is shown for selected simulation scenarios described in Fig 2B, 2C and 2D. Plateaus of *M(t)* indicate periods of isolation, with the level of the plateau indicating how much ancestry has merged before. (B) Schematic coalescence in the *Split-with-archaic-admixture* scenario. In this scenario, a fraction *1-α* of lineages sampled from the two extant populations merges at time *T*, and the rest, of proportion *α* merges as time *T*_*a*_. (C) For the *split-with-archaic-admixture* scenarios (with and without bottleneck), we can use the level of the plateau in *M(t)* to estimate *1-α*, and thus *α*. The level of the plateau is measured at time *t =* 300kya.

We also find two phases of migration for the *split-with-archaic-admixture* scenario, but this time with one phase around time *T*, and another one around the time of divergence of the archaic population *T*_*a*_ (Fig 2C). To understand this, consider how lineages in the two extant populations merge into each other (Fig 3B). One fraction *1-ɑ* will merge into each other at the population split time *T*, as in the *clean-split* scenario. The other fraction, *ɑ*, will merge back only at the deep divergence time of the archaic lineage. These two merge events correspond to the two pulses we observe in Fig 2C—one at *T* and the other at the divergence time with the archaic population, *T*_*a*_. Note that unlike in the above *split-with-migration* case, here there is no signal at the time of introgression, but only at the two split times. Inferring these two migration pulses in the presence of archaic admixture is robust to demographic events, as we show with the *archaic-admixture-with-bottleneck* scenario (Fig 2D), in which we introduced a bottleneck in one of the two extant population branches, similar in strength to the one observed in Non-African populations around 60 thousand years ago (kya) [4]. We find, however, that in the presence of a bottleneck the second pulse is a bit more recent than expected (here at 1 million years ago).

We can analyze these multiple phases of migration in a more quantitative way, by using the cumulative migration probability, *M(t)*, as introduced above. *M(t)* monotonically increases from 0 to 1 in all scenarios, exhibiting plateaus with gradient zero at times of no migration, and positive gradients in periods of migration (Fig 3A and S2 Fig). The level of these plateaus is indicative of how much ancestry has already merged at this point in time. Consider first the *split-with-migration* series (Fig 3A top panel), for which *M(t)* exhibits a plateau between the two migration pulses, at a level that corresponds to the amount of ancestry that has merged through the migration event. For this scenario, based on the simulated post-split migration rate between the two populations, we expect this plateau to be at around 0.64 (following the calculation in formula (64) in S1 Text). We find it to be higher than that, around 0.75, which we discuss further below. Consider now a scenario with archaic admixture (Figs 2C, 2D and 3A middle and bottom panels). At time *T*, at which both extant populations merge into each other, the cumulative migration probability reaches a plateau at a level around *1-ɑ*, reflecting the fact that a proportion *ɑ* has not yet merged at point *T*, but is separated by a deeply diverged population branch. Only at time *T*_*a*_, this branch itself merges into the trunk of the extant populations, thereby increasing *M(t)* from *1-ɑ* all the way to 1. Based on this rationale, we can use visible plateaus in *M(t)* to estimate fractions of archaic or otherwise deep ancestry. Indeed, this rationale leads to estimates of archaic admixture proportions in our simulations which are accurate and robust to bottlenecks for rates of *ɑ* up to about 20%. For larger introgression rates, we find our estimates to be slightly underestimated. We attribute this to MSMC’s tendency to “overshoot” changes in coalescence rates, as can be seen in the relative cross-coalescence rates for larger values of alpha (S2C and S2D Fig), which causes the level of the plateau in *M(t)* to be higher than *1-ɑ*, and hence ɑ to be underestimated. This is also the reason for the above-mentioned overestimation of the plateau in the *split-with-migration* scenario (Fig 3A top panel). This effect is more severe in the presence of a bottleneck (Fig 3C, blue curve) than without a bottleneck. Importantly, though, we find no evidence that *M(t)* exhibits plateaus below 1 in the absence of true deep ancestry, so this method can be considered conservative for detecting deep ancestry.

MSMC-IM also fits population sizes, which can be compared to the raw estimates from MSMC, i.e. to the inverse coalescence rates within population 1 and 2, respectively (see S1 Text for some non-trivial details on this comparison). We find that estimates for *N*_*1*_*(t)* and *N*_*2*_*(t)* are in fact close to the inverse coalescence rates, with some deviations seen in deep times, and in cases of archaic admixture. The latter is expected, given that estimated coalescence rates from MSMC2 capture both population size changes and migration processes, while in MSMC-IM these two effects are separated (S3 Fig).

### Deep ancestry in Africa

**Fig 4:**
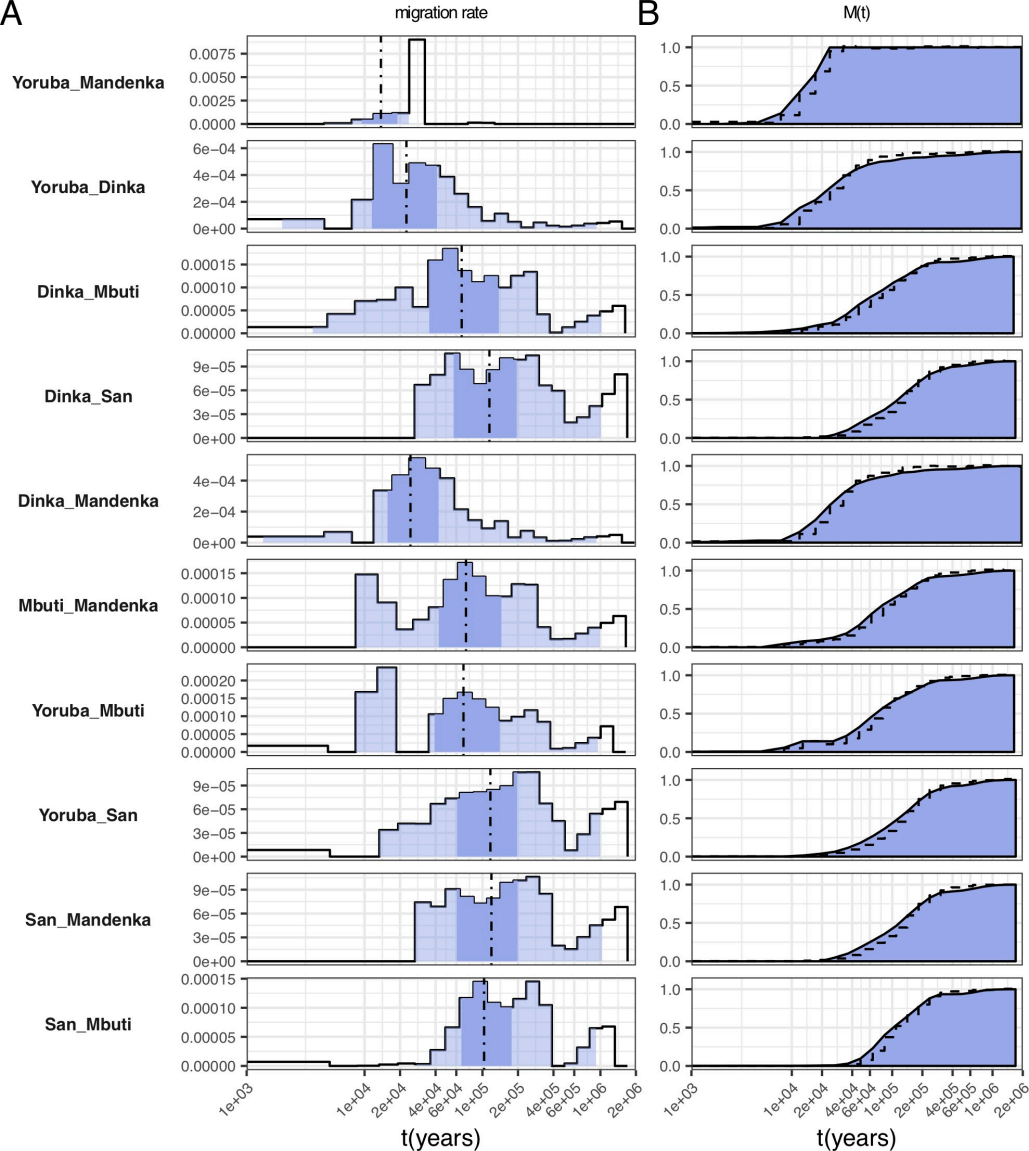
Migration rate profiles for selected pairs of African populations. (A) Migration rates. Dashed lines indicate the time point where 50% of ancestry has merged, and shading indicates the 1%, 25%, 75% and 99% percentiles of the cumulative migration probability (see Fig 2). (B) Cumulative migration probabilities *M(t)*. Dashed lines indicate the relative cross coalescence rate obtained from MSMC2, for comparison. See S4 Fig for the full set of figures.

**Fig 5:**
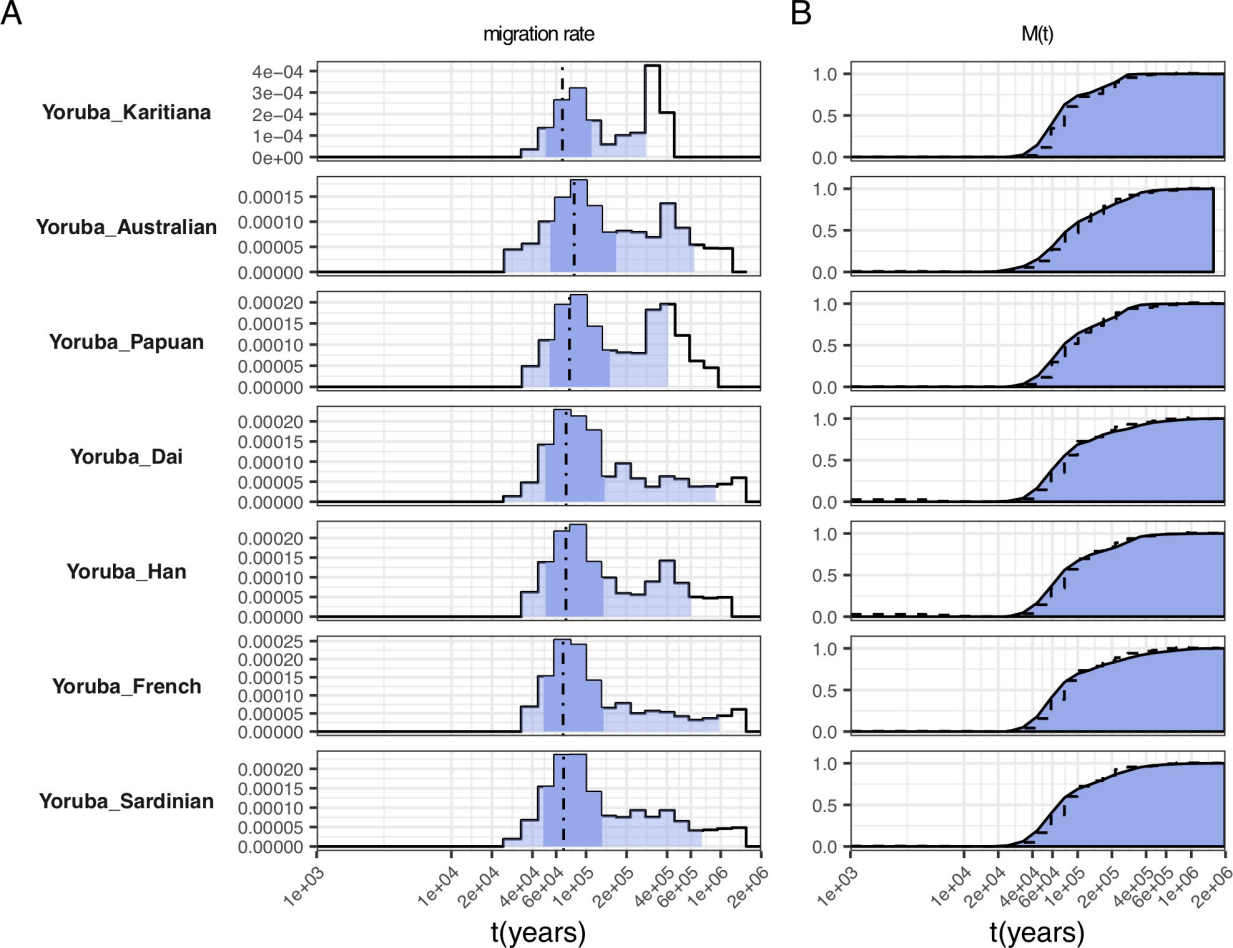
Selected migration profiles between Yoruba and 7 non-African populations. (A) Migration rates. Dashed lines indicate the time point where 50% of ancestry has merged, and shading indicates the 1%, 25%, 75% and 99% percentiles of the cumulative migration probability (see Fig 2). (B) Cumulative migration probabilities *M(t)*. Dashed lines indicate the relative cross coalescence rate obtained from MSMC2. See S4 Fig for the full set of figures.

**Fig 6:**
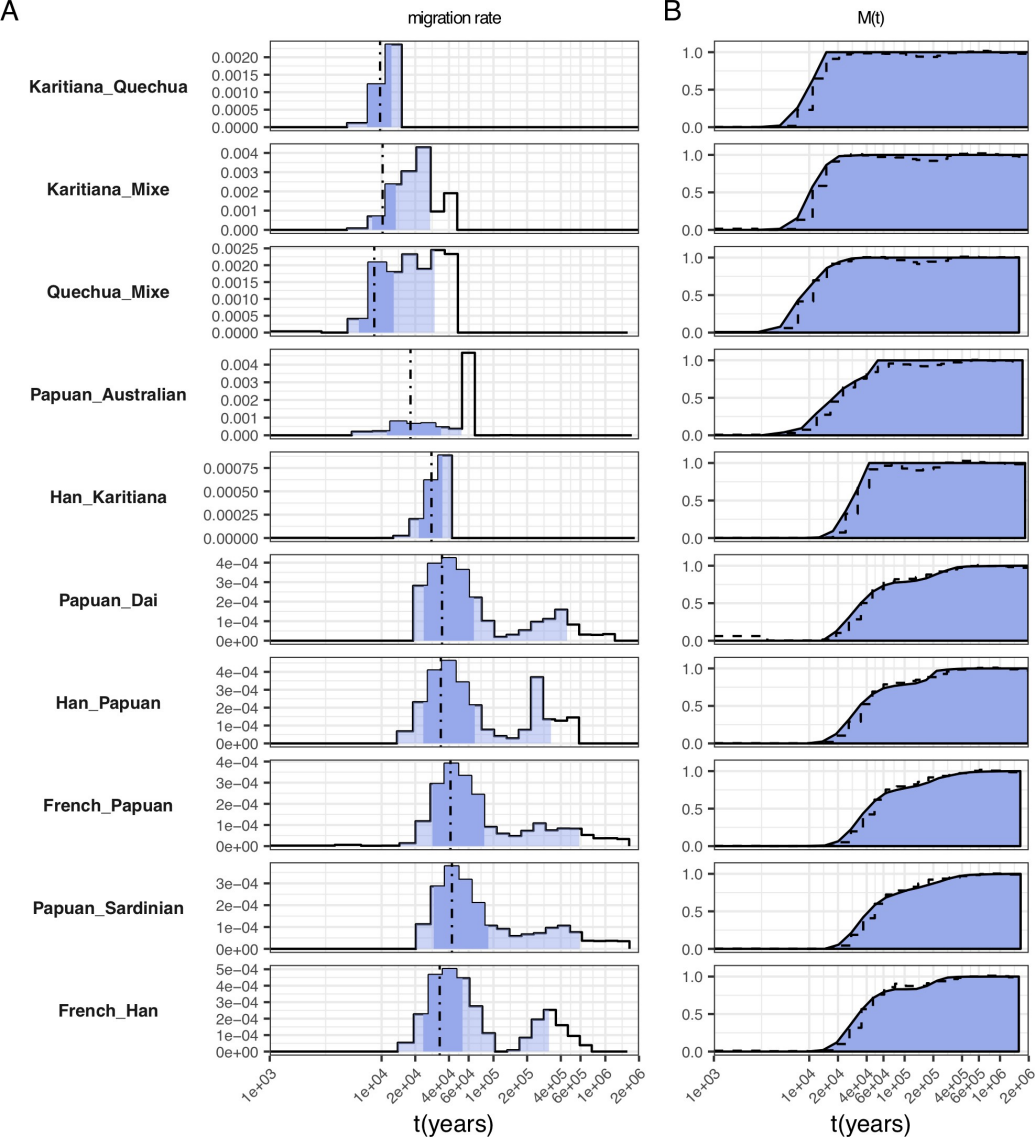
Selected migration profiles within non-African populations. (A) Migration rates. Dashed lines indicate the time point where 50% of ancestry has merged, and shading indicates the 1%, 25%, 75% and 99% percentiles of the cumulative migration probability (see panel B). (B) Cumulative migration probabilities M(t). Dashed lines indicate the relative cross coalescence rate obtained from MSMC2. See S4 Fig for the full set of figures.

**Fig 7:**
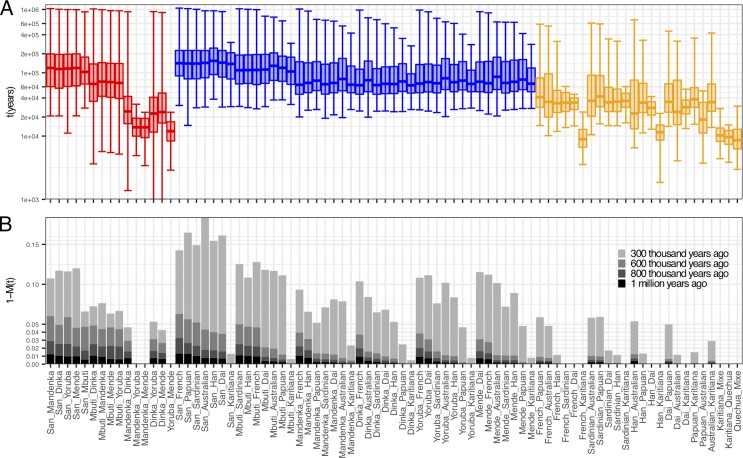
Summary profiles for divergence processes for 81 pairs of populations from 15 populations. (A) Boxes show the 25% to 75% quantiles of the cumulative migration probability *M(t)*, with bi-directional elongated error bars representing 1% and 99% percentiles. Colorcode: Red for African/African, blue for African/Non-African and orange for Non-African/Non-African pairs. (B) Barchart showing the amount of ancestry due to lineages older than 300, 600, 800kya and 1 million years ago, based on the cumulative migration probability *M(t)*.

We applied our model to 30 high coverage genomes from 15 world-wide populations from the SGDP dataset [12] (S1 Table) to analyze global divergence processes in the human past (Figs 4–6). When analyzing the resulting pairwise migration rate profiles, we find that several population pairs involving African populations exhibit by far the oldest population structure observed in all pairwise analyses. We find that in all population pairs involving either San or Mbuti, the main separation process from other populations dates to between 60-400kya, depending on the exact pair of populations (see below), but with small amounts reaching back to beyond a million years ago, as seen by the non-zero migration rates around that time (Fig 4A, S4 Fig), and the cumulative migration probability, *M(t)*, (Fig 4B) which has not fully reached 1 until beyond a million years ago. Following the interpretation of *M(t)* as discussed above with the archaic-admixture simulation scenario, we can infer that in pairs involving San or Mbuti, at least around 1% of ancestry can be attributed to lineages of ancestry that have diverged from the main human lineage beyond 1 million years ago (see also Fig 7, discussed further below). The genetic separation profile in pairs involving Mbuti and San is, beyond the extraordinary time depth, not compatible with clean population splits (as seen in simulations, Fig 2A) or simple scenarios of archaic admixture, but instead shows evidence for multiple or ongoing periods of gene flow between (unsampled) populations. Between Mbuti and other African populations except San, we find three distinct phases of gene flow. The first peaks around 15kya, compatible with relatively recent admixture between Mbuti and other African populations. The second phase spans from 60 to 300kya, reflecting the main genetic separation process, which itself looks complex and exhibits two peaks around 80-200kya thousand years ago. The third and final phase, including a few percent of lineages from around 600kya to 2 million years ago, likely reflects admixture between populations that diverged from each other at least 600kya. In pairs that include San, the onset of gene flow with other populations is more ancient than with Mbuti, beginning at around 40kya and spanning until around 400kya in the main phase, and then exhibiting a similarly deep phase as seen in Mbuti between 600kya and 2 million years ago. We confirm that this deep divergence is robust to phasing strategy (see below) and filtering (see Methods). We also replicated this signal using an independent dataset [14] (S5 Fig). An exception to these signals seen with San and Mbuti are pairs involving Karitiana, which do not exhibit such deep divergence. This is likely due to the strong genetic drift present in Karitiana, and the low heterozygosity in that population [12], which may shadow deep signals.

Apart from the deep structure seen with Mbuti and San, we find the second-most deep divergences between the West African Yoruba, Mandenka and Mende on the one hand, and French on the other (Fig 5A, S4 Fig, Fig 7 discussed further below), based on the time when M(t) reaches 99%. This might be consistent with recent findings of archaic ancestry in West-Africans [15,16], although it is not clear why the signal is primarily seen with French, and less consistently with Asian populations (Yoruba/Han have deep divergences, as well as Mende/Dai and Mandenka/Dai, but not other West-African/Asian combinations). Finally, pairwise analyses among Mende, Mandenka and Yoruba (Fig 4A, S4C, S4E and S4F Fig) exhibit a very recent migration profile, which appears to span up to about 20kya but not older, which is at odds with a recent finding of basal African ancestry present to different degrees in Mende and Yoruba [17]. However, that signal may be too weak to be detected in our method, which is based on only two individuals per population.

### Complex divergence between African and Non-African populations

Compared to the separation profiles between San or Mbuti and other populations, separations between other Africans and non-Africans look relatively similar to each other, with a main separation phase between 40 and 150kya, and a separate peak between 400 and 600kya (Fig 5 and S4 Fig). The first, more recent, phase plausibly reflects the main separation of Non-African lineages from African lineages predating the “out-of-Africa” migration event, and coinciding with the major population size bottleneck observed here (S6 Fig) and previously [3,4] around that time period. Signals more recent than about 60kya likely reflect the typical noisy spread of MSMC-estimated coalescence rate changes observed previously [4]. The second peak of migration, between 400 and 600kya likely reflects Neandertal and/or Denisovan introgression into non-Africans. The age of that peak appears slightly more recent than, although overlapping with, previous split time estimates of those two Archaic groups from the main human lineage at 550-765kya [14]. However, our simulation with archaic admixture with bottleneck (Fig 2D), shows that our model tends to underestimate the archaic split time in the presence of population bottlenecks as is the case for non-African populations [18–20]. In favor of the hypothesis that this second peak is caused by archaic lineages that have contributed to non-Africans is the fact that in all pairs of Papuans/Australians vs. Yoruba/Mende/Mandenka or Dinka, the second peak is particularly pronounced. This fits the archaic contribution hypothesis, since Papuans and Australians are known to have among all extant human populations the highest total amount of ancestry related to Neanderthals and Denisovans.

We investigated previous observations of potential ancestry from an earlier dispersal out of Africa, present in Papuan and Australian genomes [12,13,21]. Previously, one line of evidence for such a signal was based on shifts of relative cross coalescence rate curves between some Africans and Papuans or Australians on the one hand compared to curves with Europeans or East Asians on the other. With MSMC-IM we can compare these curves more quantitatively. While we were able to replicate this slight shift of relative cross coalescence rate or *M(t)* midpoint-based split times from African/Eurasian pairs to African/Australasian pairs reported in Ref. [21] using MSMC and Ref. [13] using MSMC2, we find that the estimated migration profiles of these pairs are very similar (S7 Fig), with a main separation midpoint around 70kya and a second older signal beyond 200kya, consistent with both Australasians and other Non-Africans being derived from a single genetic ancestral population without a more basal contribution to Australasians [12,13]. We conclude that the shift in the relative cross coalescence rate curve appears to be consistent with being caused by the higher amount of archaic ancestry present in Papuans and Australians. We note, however, that different separation events are not distinguishable in MSMC-IM when they are temporally close to each other, as we saw in the *split-with-migration-scenario* (Fig 2B).

### Separations outside of Africa

All separations outside of Africa are younger than separations between Africans and Non-Africans, as expected (Fig 6, S4 Fig). The deepest splits outside of Africa are seen in pairs of Papuans or Australians with other Eurasians, in which the first peak of migration is seen at 34kya, corresponding to the early separation of these populations’ ancestors from other non-African populations after the out of Africa dispersal. In these pairs we see a second peak around 300kya, likely corresponding to the known Denisovan admixture in Papuans and Australians [13,22]. This is too recent for divergence time estimates between Denisovans and modern humans [14], which again is consistent with the underestimate seen in simulations with bottlenecks. Surprisingly, we see a similar second peak between French and Han, which is consistent with cross-coalescence rate features in previous observations [4,12] but of unclear cause. Consistent with the hypothesis that the second peak seen in Australasian/Eurasian pairs corresponds to Denisovan admixture, we do not see a second peak in the migration profile between Papuans and Australians, confirming that the gene flow likely occurred into the common ancestor of Australians and Papuans [13]. The migration profile between Papuans and Australians shows a main separation between 15-35kya.

The second deepest splits in Non-African populations are seen between East Asian and European populations, which occur mostly between 20 and 60kya (cumulative migration probability midpoint at 34kya), followed by separations between Asian and Native American populations, between 20 and 40kya (midpoint at 28kya). The latter likely also reflects Ancestral North Eurasian ancestry in Native Americans [23], which is more closely related to Europeans than to East Asians, thereby pushing back the separation seen between East Asians and Native Americans. Finally, the most recent splits are seen between populations from the same continent: Dai/Han split around 9-15kya (midpoint 11kya), French/Sardinian around 7-13kya (midpoint 9kya) and within Native Americans around 7-13kya (midpoint 10kya) (Fig 6, S4 Fig).

To visualize the depth of ancestry in each population pair, we summarized all pairwise analyses by percentiles of the cumulative migration probability *M(t)* (Fig 7). Largely, Non-African pairs (orange) have their main separation phase, with the cumulative migration probability between 25% and 75%, between 20 and 60kya, with some more recently diverged pairs within continents. In contrast, African pairs (red) have their main phase largely between 60 and 200kya, with some notable exceptions of more recently diverged populations, and with the notable tail (99% percentile) up to 1 million years and older. Between Africans and Non-Africans, divergence main phases are largely within a similar window of 60-200kya as in African pairs, with three notable groups: divergence of Non-Africans from San falls between 80-250kya, from Mbuti between 70-200kya, and from other Africans between 50-150kya. To highlight the amount of ancestry contributed asymmetrically to one of the two populations from unsampled populations that diverged from the human lineage in the deep past (so-called archaic lineages), we show the distance of the cumulative migration probability from 1, 1-*M(t)*, at different deep time points (Fig 7B). As described above, the deepest signals are seen in pairs involving San or Mbuti, reaching 3% of ancestry contributed from lineages that diverged at least 800kya, and around 1% of ancestry from lineages that diverged at least 1 million years ago. Similarly deep levels are seen in specific pairs involving French, in combination with the West African Mende, Mandenka and Yoruba and the East African Dinka, and for pairs Mende/Dai and Mandenka/Han, as discussed above.

### Robustness to phasing and processing artifacts

MSMC2 (like MSMC) requires phased genomes for cross coalescence rate estimation, and we therefore rely on statistical phasing within the SGDP dataset, for which different strategies are possible. To compare the effect of selecting such phasing strategy, we generated phased datasets using eight different phasing strategies with three phasing algorithms (SHAPEIT [24], BEAGLE [25], EAGLE [26]). We included genotype calls from 12 individuals with previously published physically phased genomes [12] and then used those genomes to estimate the haplotype switch error rate. Among eight phasing strategies, SHAPEIT2 [24], without the use of a reference panel, but including information from phase-informative reads [27], resulted in the lowest switch error rate per kb (and per heterozygous site; S8 Fig). Overall, switch error rates are higher in African populations, likely due to lower linkage disequilibrium, higher heterozygosity and relatively limited representation in the SGDP. To test how sensitive MSMC-IM is to different phasing strategies, we tested four phasing strategies on four different pairs of populations with evidence for extremely deep ancestry (Methods). We find that the migration profile from MSMC-IM is very similar for different phasing strategies. In particular, we find that the very deep signal seen in population pairs involving San and Mbuti is reproduced with different phasing strategies with and without a reference panel (S9A Fig). In a similar way, we confirmed the robustness of that signal with respect to choosing different filter levels (S9B Fig) and with respect to removing CpG sites, which are known to have elevated mutation rates (S9C Fig). We also explored to what extent switch errors affect our estimates using simulated data (S10 Fig), and confirmed robustness with respect to variation in recombination rates, which are assumed to be constant along the genome within MSMC2 but vary in reality (S11 Fig). Finally, to test internal consistency, we tested how well MSMC-IM was able to infer back its own model. We used the estimated migration rates and population sizes from eight population pairs (see Methods), and simulated genomic data under their inferred models. As shown in S12 Fig, the estimated migration patterns from the simulated and the real data are indeed very similar, including the deep signals seen in pairs with San and Mbuti.

Given the superiority of the read-aware phasing strategy with SHAPEIT without a reference panel [27,28] (S8 Fig), we used this method in all of our main analyses. However, even with this phasing strategy, the switch error rate is high in populations that are not well represented in the dataset. In case of indigenous Australians, the phasing quality is among the worst in the dataset (S8 Fig), arguably because the SGDP dataset contains only two Australian individuals (compared for example to 15 Papuans). To improve phasing in Australians specifically, we generated new high coverage genomic data for one of the two Australians in the SGDP dataset using a new library with longer read-pair insert sizes (see Methods). Using these additional reads reduced the switch error rate from 0.038/kb to 0.032/kb. (S8 Fig, blue isolated dot for Australian3). We ran MSMC2 on the long-insert Australian data, as well as the standard phased data, combined with one diploid genome from each of the other world-wide populations analyzed in this study. The inferred migration profiles from MSMC-IM (S13 Fig) for Non-African population pairs involving the long-insert phased Australian genome do not seem to be affected by the phasing method (S13 Fig). The migration profile from pairs of Africans versus the long-insert phased Australian tend to be slightly younger, but also show deeper structure in Dinka/Australian, compared to the same pair using the *shapeit_pir* phasing method, which uses phase-informative sequencing reads to improve phasing accuracy (Methods). Note that these migration rate densities exhibit more noise than the ones used in our main analysis (S4L Fig), since they are based on only one individual per population, while the main analyses are based on two individuals per population. The main separation between Papuan and Australian remains at 15-35kya, as shown in the migration profile from both phasing strategies, very close to the estimates from 8 haplotypes in the main analysis (S4L Fig), and earlier than the previous estimates of 25-40kya [13].

Similar to the procedure introduced for PSMC [3], we use a block-bootstrap approach to assess statistical uncertainty of our method. We find that there is very little uncertainty around MSMC-IM’s migration rate estimates (S14 Fig) based on these bootstrap-estimates. This should be taken with caution, though, since the bootstrap is only able to address the uncertainty caused by randomness in the data, not by systematic biases. We know that MSMC typically “smears out” sudden changes in coalescence rates, which is due to the wide variance in local estimates of coalescence times, and this type of error is not revealed by the bootstrap. It does, however, give high confidence to specific results, such as estimates of archaic ancestry between 1 and 20% as seen in Fig 3C. According to our bootstrap test (S14 Fig), the cumulative migration probability M(t) does hardly vary at all in bootstrap replicates, so estimates of deep ancestry fractions such as the ones shown in Fig 3C and Fig 6B for real data, are very accurate.

## Discussion

We have presented both a novel method MSMC-IM for investigating complex separation histories between populations, and an application of that method to human genomes, revealing new insights into the complex separations and deep ancestry in African populations. MSMC-IM extends MSMC2 by fitting an IM model to the estimated coalescence rates, which allows us to characterize the process of population separation via a continuous migration rate through time. In contrast to the established approach of using the relative cross coalescence rate directly from MSMC2, our new approach interprets coalescence rates more quantitatively. In a recent study a similar approach has been used to fit an IM model to PSMC estimates to estimate population split times and post-split migration rates in a more strictly parameterized model [29]. We found here that a continuous IM model without an explicit split time better fits the estimated coalescence rates from MSMC2, which are continuous themselves and thus lead to a more gradual concept of population separation. This absence of an explicit population split time distinguishes our approach from many previous models [5,8,9] and allows us to detect new signals of temporal population structure without specifying population phylogenies or admixture graphs from prior knowledge or via inference.

A showcase example for such new insights are the traces of extremely deep population structure seen in our analysis of African population pairs. The fact that San and Mbuti exhibit the deepest branches in the human population tree is itself not surprising given previous analyses [30–34], but the extraordinary time depth displayed in this analysis has to our knowledge not been reported before. This deep structure—albeit only making up 1% of ancestry—is far older than the oldest attested fossil records of anatomically modern humans, considering the East-African fossils of Omo Kibish and Herto 160-180kya [34–36] and the skull from Jebel Irhoud recently re-dated to around 300kya [37]. Any admixture from an archaic population that diverged from the main human lineage more than 600kya would produce such a signal. This is the case, for example, for the so-called “super-archaic” population that was inferred to have admixed into Denisovans [14] and was estimated to have diverged from the lineage leading to modern Humans, Neanderthals and Denisovans between 1.1 and 4 million years ago. Given this finding outside of Africa, it is perhaps not surprising that such deep archaic population structure existed also in Africa.

However, our signal of archaic population structure in Africa reveals more complexity than expected under the standard model of archaic introgression, in which two divergent populations admix with each other, creating a distinct pattern of deep ancestry in the genomes of the target population. Detecting such patterns in the genome would require a sufficient sequence divergence between non-introgressed and introgressed genomic segments and sufficiently long introgressed segments (as detected by the S* statistic or extensions of it [15,16]). This is the case if the majority of ancestry between the two intermixing species has been isolated for hundreds of thousands of years, with a relatively recent introgression time (comparable to the time of the Neanderthal introgression). Such a scenario would then be seen as a bimodal pattern in the migration profile, as shown in our simulations (Fig 2C). What we see, however, in the migration profiles between San and Mbuti with other African populations, is not a bimodal pattern, but a more continuous distribution. This would emerge under a model of repeated isolation and partial admixture of two or more archaic species or populations that exist in parallel for a long time. Under such a scenario, genomes are not a two-way mixture between introgressed and non-introgressed regions, but a mosaic of ancestry lines merging at a range of different split times. Since much of the introgression would then be attributed to very ancient events, these segments would be too short for methods such as S* to be detected as archaic ancestry, which may be the reason why the deep signals reported here have not been reported before for San and Mbuti, in contrast to Non-Africans and West Africans [15,16].

While the continuous model in MSMC-IM adds significantly to previous approaches to estimating population separations, one drawback is that it is currently limited to only two populations at a time. While this limit is partially technical—MSMC2 cannot be scaled to arbitrary numbers of genomes—the more severe problem is a conceptual one. It is not obvious how to use the concept of continuous-time migration rates and non-sharp population separations to more than two populations. One possibility are graph models, as they are used in admixture graphs [38], but it is unclear how to make such models fully continuous, as is our current migration rate and cumulative migration probability for two populations. An important direction for future work is to achieve a generalization of the continuous concept of population separation to multiple populations, which might help to better understand and quantify the processes that shaped human population diversity in the deep history of our species.

## Materials and methods

### MSMC2

MSMC-IM is based on MSMC2 (first described and used in Ref. [13]) as a method to estimate pairwise coalescence rates from multiple genome sequences. The MSMC2 method is summarized in a self-contained way in S1 Text. MSMC2 is similar to MSMC [4], but instead of analyzing multiple genomes simultaneously modelling the first coalescence event, it uses the pairwise model in sequence on all pairs of haplotypes to obtain a composite likelihood of the data given a demographic model. The demographic model itself (consisting of a piecewise constant coalescence rate) is then optimized via an Expectation-Maximization algorithm similarly to MSMC and PSMC [3]. For cross-population analyses, we use MSMC2 to obtain three independent coalescence rate estimates: two coalescence rates through time within each population, named *λ*_*11*_*(t)* and *λ*_*22*_*(t)*, respectively, and one coalescence rate function for lineage pairs across the population boundary, named *λ*_*12*_*(t)* (S1 Text).

### MSMC-IM model

MSMC-IM then fits a two-island model with time-dependent population sizes *N*_1_(*t*) and *N*_2_(*t*), and a time-dependent continuous symmetric migration rate *m(t)* to the estimated coalescence rates, which essentially is a re-parameterization from the triple of functions {*λ*_*11*_*(t)*, *λ*_*12*_*(t)*, *λ*_*22*_*(t)*} to a new triple of functions {*N*_*1*_*(t)*, *N*_*2*_*(t)*, *m(t)*} (S1 Text). To fit the island-model to the coalescence rates, we first use the coalescence rates to compute a probability density for times to the most recent common ancestor (tMRCA), as illustrated here for rate *λ*_*11*_*(t)*:

$$P^{MSMC}(t|s_{0}=S_{11})=\lambda_{11}(t)e^{-\int_{0}^{t}\lambda_{11}(t\prime )dt\prime }$$

Here, *S*_11_ denotes the starting state where both lineages are present in population 1. We then use an approach by Hobolth et al 2011 [39] to compute this density for the three starting states *s*_0_ = {*S*_11_,*S*_12_,*S*_22_} under an IM model, denoted *P*^*IM*^(*t*|*s*_0_), using exponentiation of the rate matrix of the underlying IM-Markov process that governs the state of uncoalesced and coalesced lineages in two populations connected by a time-dependent migration rate (see S1 Text). The fitting process of the IM model to the probability density computed from MSMC2 is done by minimizing the Chi-square statistics:

$$\chi^{2}=\sum_{i=1}^{n_{T}}\left[\sum_{s_{0}\in \{S_{11},S_{12},S_{22}\}}\frac{{\left(P^{IM}(t_{i}|s_{0})-P^{MSMC}(t_{i}|s_{0})\right)}^{2}}{P^{MSMC}(t_{i}|s_{0})}+\beta_{1}\int_{0}^{\infty }m(t_{i})\;dt+\beta_{2}{\left(\frac{N_{1}(t_{i})-N_{2}(t_{i})}{N_{1}(t_{i})+N_{2}(t_{i})}\right)}^{2}\right]$$

where *n*_*T*_ denotes the number of time segments, and the t_i_ denote the boundaries of the discrete time segments. The second and third term in the formula are regularization terms to avoid overfitting, with *β*_1_ restricting migration rates and *β*_2_ pushing the two population sizes *N*_*1*_*(t)* and *N*_*2*_*(t)* close to each other. The strength of this regularization can be controlled via a user-defined parameter in our program. We sum over Chi-square statistics over *n*_*T*_ time intervals with *i* representing the time index in the formula. For the three simulation scenarios and all pairs of real data, we used a regularization value of *β*_1_ = 10^−8^, *β*_2_ = 10^−6^. Regularization is necessary because the reparameterization introduced by MSMC-IM overspecifies the model at times when the two populations are fully merged. For that same reason, we plot estimated migration rates in all figures only up to a value of *M(t)* = 0.999, since migration rate estimates beyond that point are essentially arbitrary, as lineages have already been fully randomized between the two populations. We also restrict the estimated population sizes to 10,000,000 in practice.

We implemented the MSMC-IM model as a python command line utility that takes the MSMC or MSMC2 output files as input. The program is available at: https://github.com/wangke16/MSMC-IM.

### Simulations

We used *msprime* [40] for all simulations in this paper. In the three series of simulation scenarios mentioned above, we simulated four diploid genomes composed of 22 chromosomes each of length 100Mbp from two populations, assuming a constant population size 20,000 for every population. The recombination rate we used here is 10^−8^ per generation per bp, and the mutation rate is 1.25×10^−8^.

In the zig-zag simulation (S1 Fig), we simulated a series of exponential population growths and declines for two, four and eight haplotypes, each changing between 3,000 and 30,000 in exponentially increasing time intervals, with the same simulation parameters as specified in Ref. [4] and Ref. [3] to ensure comparability with these previous publications. In particular, this simulation involved a lower recombination rate (0.3×10^−8^) than the main simulations, justified in Ref. [4] as the inferred recombination rate from real data using PSMC’. The reason for it being lower than the true recombination rate (close to 10^−8^, as used in the main simulations above), is that MSMC (and MSMC2) infers an “effective recombination rate”, which is a non-trivial average over the variable recombination landscape across the human genome.

We also conducted a number of simulations based on MSMC-IM inference from real data (S12 Fig). We took the estimates on migration rates and population sizes from MSMC-IM (S2 Table) for eight pairs of worldwide populations (San/Mbuti, San/Dinka, San/French, Mbuti/French, Yoruba/French, Yoruba/Papuan, French/Han, Papuan/Australian), as the input parameters in our simulation, and simulated 2.2Gb genomes on 8 haplotypes for each case. The recombination rate we used here is 10^−8^ per generation per bp, and the mutation rate is 1.25×10^−8^.

### Processing genomic Data

For the results shown in Figs 4–7, we used 30 high coverage genomes from 15 cross-continental modern populations in the SGDP dataset [12], with two diploid genomes from each population for running MSMC2 and MSMC-IM (S1 Table). Only the autosomal genome was used for this analysis. We ran pairwise analyses for 13 populations (excluding Quechua and Mixe) and pairwise comparisons within three native American populations (81 population pairs in total). We downloaded the *cteam-lite* dataset of from the website: http://reichdata.hms.harvard.edu/pub/datasets/sgdp/, in the *hetfa*-format where all sites are represented by an IUPAC encoding representing diploid genotypes, along with individual masks recording the quality of the genotype calls. We converted the *hetfa* mask files (.ccompmask.fa.rz) to zipped bed format though two steps: first we uncompressed the *hetfa* mask files using “*htsbox razip -d -c*” (https://github.com/lh3/htsbox), and then converted the uncompressed mask files (.ccompmask.fa) to zipped bed format by an in-house python script adapted from the *makeMappabilityMask*.*py* script in *msmc-tools* (www.github.com/stschiff/msmc-tools). The *cteam-lite* masks encode quality using an integer-range from 0 to 9 (reflecting increasing stringency) and “N” to represent missing data. For our analysis, we included all sites that were non-missing, i.e. have a minimum quality level of 0.

Following the processing introduced in PSMC [3] and MSMC/MSMC2 [4], beyond the individual masks we also use a universal mask to reflect overall mappability and SNP calling properties along the human genome. We used the universal masks defined in Supplementary Info 4 from Ref. [12] (and available for download at https://github.com/wangke16/MSMC-IM/tree/master/masks) as additional negative masks denoting genomic regions to be filtered out.

Beside the genome-wide mask files for each individual, we obtained variant data as made available on the SGDP project website (https://sharehost.hms.harvard.edu/genetics/reich_lab/sgdp/phased_data/). Due to the specifics of how that dataset was generated, only segregating sites at positions where the Chimpanzee reference genome has non-missing data are included. To balance this missingness based on the Chimpanzee reference genome for MSMC, we included an additional mask in our preprocessing, which reflected non-missing regions in the Chimpanzee reference sequence. For others to reproduce our analysis, we provide this chimp mask on the MSMC-IM *github* repository (https://github.com/wangke16/MSMC-IM).

We phased the data using SHAPEIT2 (v837) [24], Beagle4.0 (r1399) [25] and EAGLE2 (version 2.3) [26]. We first phased the data using each algorithm both with and without a reference panel (here we used the 1000 Genomes Phase 3 reference panel as recommended in the Shapeit2 documentation). When using a reference panel, all three methods are only able to phase sites that are represented in the reference panel. Therefore, we removed sites not in the reference panel, phased, adding the removed sites back as unphased, and then ran a second round of phasing using Beagle4.0 and the “usephase = true” option, which allows us to phase the unphased sites in data that is already partially phased. Finally, we also phased using SHAPEIT2 without a reference panel, but using the read-aware phasing strategy [27]. This uses the fact that two SNPs found on the same (paired) read must be in phase. The switch error of each of these phasing strategies, evaluated by comparison with the experimentally phased data generated for the same samples [12] is shown in S8 Fig.

Finally, we generated a long-insert library from one of the two Australian DNA samples analyzed in SGDP [12], with a median insert size of 3.3kbp. These data are available at the European Nucleotide Archive under accession number ERX1790596 (https://www.ebi.ac.uk/ena/data/view/ERX1790596). We used this data to improve the phasing quality for this Australian individual. As shown in S8 Fig, this strategy indeed reduced the switch error rate for this Australian individual from 0.036/kb to 0.032/kb.

### Running MSMC-IM

Unlike MSMC, which reports these three rates in a single analysis step, in MSMC2 we run the three estimations for *λ*_*11*_*(t)*, *λ*_*12*_*(t)* and *λ*_*22*_*(t)* independently from each other, using a different selection of haplotype pairs in each case. We base most of our analyses on 4 diploid individuals (unless indicated otherwise), for which we prepared joint input files for each chromosome, consisting of 8 haplotypes each. We then chose the pairs to be analyzed using the “-I” option in MSMC2. For coalescence rate *λ*_*11*_*(t)*, we used “-I 0,1,2,3”, which instructs MSMC2 to iterate through all six possible haplotype pairs among the four haplotypes from the first population. Likewise, to estimate *λ*_*22*_*(t)*, we used “-I 4,5,6,7”. Finally, to obtain estimates of the coalescence rates across populations, *λ*_*12*_*(t)*, we used “-I 0–4,0–5,0–6,0–7,1–4,1–5,1–6,1–7,2–4,2–5,2–6,2–7,3–4,3–5,3–6,3–7”, iterating through all sixteen possible haplotype pairings between the four haplotypes in each population. MSMC-IM requires a single input file containing all three coalescence rate estimates, similar to the output generated by the original MSMC program. A script *combineCrossCoal*.*py* is provided on the *msmc-tools github* repository (http://www.github.com/stschiff/msmc-tools), to generate the combined output file from the three output files of the three MSMC2 runs for a pair of populations.

With the combined MSMC2 output as input, we run MSMC-IM model by “***MSMC_IM*.*py pair*.*combined*.*msmc2*.*txt***”. Also, the time pattern needs to be specified, which is by default 1*2+25*1+1*2+1*3 as the default in MSMC2. In the output, MSMC-IM will rescale the scaled time in MSMC2 output by mutation rate 1.25e-8 into real time in generations, and report symmetric migration rates and *M(t)* in each time segment.

### Robustness tests

Phasing Strategy: We tested the robustness of our findings by applying four different phasing strategies—*beagle*, *shapeit*, *shapeit_ref_all* to *shapeit_pir* to four pairs of populations in the SGDP dataset (San/Mbuti, San/Yoruba, San/French, Mbuti/French). Here, *beagle* and *shapeit* denote phasing with no reference panel, *shapeit_ref_all* denotes phasing with a reference panel (1000 Genomes phase 3, with sites not in the reference panel phased with Beagle) and *shapeit_pir* denotes no reference panel but including phase-informative reads (S9 Fig).

Filtering: We explored the impact of mask filtering levels using San/French and Mbuti/French in the SGDP dataset, by varying the stringency of the filtering between levels 0, 1, 3, 5 (S9 Fig).

CpG islands: We conducted San/French and Mbuti/French runs with removed CpG sites. For this, we generated a mask including all positions of Cytosines and Guanines in CpG dinucleotides, Thymines in TpG dinucleotides, and Adenosines in CpA dinucleotides in the human reference genome hg19, and used those positions as negative mask when preparing the MSMC input files. This mask can be found in the *github* repository (https://github.com/wangke16/MSMC-IM).

Simulated switch errors: To explore the impact of switch errors, we added artificial switch errors at rates ranging from 5e-6 to 5e-4 per base pair in four different simulation scenarios—the *clean-split* scenario at 75kya, the *split-with-migration* scenario at 75kya, the *split-with-archaic-admixture* scenario at proportion 5%, the *split-with-archaic-admixture-bottleneck* at proportion 5%. As shown in S10 Fig, we found that the impact of switch errors on MSMC-IM’s estimates is negligible up until switch errors of rate 5e-5.

Simulations with variable Recombination rates: In the four simulation scenarios selected above we simulated variable recombination rates using a human genetic map with variable recombination rates along the genome downloaded from (ftp://ftp.ncbi.nlm.nih.gov/hapmap/recombination/2011-01_phaseII_B37/genetic_map_HapMapII_GRCh37.tar.gz). As shown in S11 Fig, MSMC-IM’s estimates from using a real genetic map are consistent with estimates from using constant recombination rates.

Bootstrapping: We applied a block-bootstrap, similar to the approach described in ref. [3] to six pairs in the SGDP dataset (San/Mbuti, San/Dinka, Yoruba/French, French/Han, Yoruba/Papuan, Papuan/Australian) with 20 replicates for each (S12 Fig).

Independent Dataset: We tested our approach on 12 populations (24 genomes) from another dataset [14], which consists of different genomes available from http://cdna.eva.mpg.de/neandertal/altai/ModernHumans/. This dataset was processed independently using the pipeline in the *msmc-tools github* repository (http://www.github.com/stschiff/msmc-tools) i.e. SNPs and masks generated using *samtools* and *bamCaller*.*py*, with statistical phasing by *SHAPEIT2* with the 1000 Genomes reference panel, leaving sites not present in the reference panel as unphased. Results between the two datasets are very similar, with some differences observed in relation to highly drifted populations like Karitiana.

## Supporting information

S1 Fig MSMC and MSMC2 population size estimates from simulated data. To test population size inference capabilities of MSMC (A) and MSMC2 (B) applied to two, four and eight haplotypes, we simulated a series of exponential population growths and declines, each changing the population size by a factor ten. The true population size is shown as dark solid line. Compared to MSMC, MSMC2 recovers the population size well, and the resolution in recent times increases with the number of haplotypes. With two haplotypes, MSMC2 infers the population history from 10kya to 3 million years, whereas, with four haplotypes and eight haplotypes the resolution in recent times is extended to 3kya and 1kya years ago respectively.
(PDF)

S2 Fig Cumulative migration probabilities from four simulation scenarios. This figure shows the same results as Fig 2, but showing M(t) instead of m(t). The scenarios are (A) the *Clean-split* scenario. (B) the *Split-with-migration* scenario, and (C) the *Split-with-archaic-admixture* scenario. (D) the *Split-with-archaic-admixture-and-bottleneck* scenario. For panel (C) and (D), we show results with alpha ranging from 0 to 1, instead of between 0 to 20% shown in Fig 2. The relative CCR is shown in step-wise dashed lines to be compared with M(t).
(PDF)

S3 Fig Population size estimates from MSMC2 compared to MSMC-IM: We simulated *N*_*1*_*(t)* and *N*_*2*_*(t)* as constant 20,000 in top three different simulation scenarios, and simulated a severe bottleneck in *N*_*2*_*(t)* with a factor 30 between 40-60kya in the bottom simulation scenario. The split time *T* is 75kya in all four cases, and all other parameters are the same as in Fig 2 and as indicated. As shown, the MSMC-IM estimates for *N*_*1*_*(t)* and *N*_*2*_*(t)* are close to the inverse coalescence rates, with relatively small effects caused by the migration rate in MSMC-IM which is absent from MSMC2.
(PDF)

S4 Fig **Pairwise migration profiles for 13 worldwide populations**, involving San (A), Mbuti (B), Mandenka (C), Dinka (D), Yoruba (E), Mende (F), French (G), Sardinian (H), Han (I), Dai (J), Papuan (K), Australian (L), Karitiana (M). The relative CCR is shown in step-wise dashed lines to be compared with M(t). *See separate joint PDF file*.
(PDF)

S5 Fig Migration profile of an independent dataset. Here we have analyzed 12 worldwide populations from Prüfer et al (2014) with independent data processing as described in Methods: San (A), Mbuti (B), Mandenka (C), Dinka (D), Yoruba (E), French (F), Sardinian (G), Han (H), Dai (I), Papuan (J), Australian (K), Karitiana (L). The relative CCR is shown in step-wise dashed lines to be compared with M(t). *See separate joint PDF file*.
(PDF)

S6 Fig Estimated population sizes from MSMC2 for 15 worldwide populations. We show the estimates from MSMC using 8 haplotypes/4 individuals per population from the SGDP dataset.
(PDF)

S7 Fig Testing for potential multiple out-of-Africa separations. Here we show analyses on the divergence of Papuans and Australians from Africans vs. other Non-African populations from Africans. We show the cumulative migration probability *M(t)* in (A), and the migration rate *m(t)* (B) for pairs of populations of Yoruba, Dinka and San with one non-African population as indicated.
(PDF)

S8 Fig Switch error rates from eight phasing strategies. *beagle* and *beagle_ref_all* denote BEAGLE phasing without and with reference panel (here and below denoting the 1000 Genomes Phase 3 reference panel). *eagle* and *eagle_ref_all* represent EAGLE phasing without and with reference panel. *shapeit* and *shapeit_ref_all* represent SHAPEIT phasing without and with reference panel. *shapeit_pir* represents SHAPEIT phasing with phase-informative reads. *shapeit_pir_extra* represents SHAPEIT phasing with long-insert-size reads as additional phase informative reads, which was applied to B-Australian-3 only. See Methods for details.
(PDF)

S9 Fig Impact of phasing and processing artifacts. We show (A) the impact of the phasing strategy using San/Mbuti, San/Yoruba, Mbuti/French and San/French as examples, (B) the impact of the filtering level for generating individual masks using San/French and Mbuti/French as example, and (C) the impact of removing CpG sites using San/French and Mbuti/French as example. See caption to S8 Fig for a description of the four phasing methods shown in (A).
(PDF)

S10 Fig Impact of switch errors on simulated data. Here we selected the same four simulation scenarios used in S3 Fig, and added phasing switch errors ranging from 5e-6 to 5e-4 per base pair. The overall migration profiles remain relatively consistent for error rates between 5e-6 and 5e-5, with strong effects seen with rates higher than 5e-5, shifting the migration profiles towards older times. (A) Clean split at 75kya. (B) Split at 75kya with symmetric migration between 10-15kya. (C) Split at 75kya with archaic admixture at 5%. (D) Split at 75kya with archaic admixture at 5% and bottleneck in one population.
(PDF)

S11 Fig Impact of recombination rate on simulated data. Applying the same four simulation scenarios used in S3 Fig, we here used the genetic map estimated for the human genome (i.e. variable recombination rate across genome) instead of a constant recombination rate. Red lines represent our estimates from using a constant recombination rate 10^−8^ per generation per bp. (A) Clean split at 75kya. (B) Split at 75kya with symmetric migration between 10-15kya. (C) Split at 75kya with archaic admixture at 5%. (D) Split at 75kya with archaic admixture at 5% and bottleneck in one population.
(PDF)

S12 Fig Migration profile on simulated pseudo-SGDP genomes. Green lines show the estimates we got from SGDP data for pairs shown on the left (as shown in S4 Fig), which are used as input parameters for the simulation. Red lines show the estimates from applying MSMC-IM on the simulated data. (A) Migration rates *m(t)*. (B) Cumulative migration probabilities *M(t)* and relative cross-coalescence rates.
(PDF)

S13 Fig Impact of long-insert phasing on Australian population separation inferences. *M(t)* in quantiles is summarized here between a single Australian and a single individual from worldwide populations. Boxes show the 25% to 75% quantiles of *M(t)*, with bi-directional elongated error bars representing 1% and 99% percentiles. Red color represents the data phased using long-insert reads. Green color represents the standard phased dataset.
(PDF)

S14 Fig Bootstrap tests. As shown in (A) migration rate m(t) and (B) Cumulative migration probability *M(t)*, the overall inferred profile for each pair is rather consistent across 20 replicates.
(PDF)

S1 Table Analyzed samples and population labels from the SGDP dataset. (XLSX)

S2 Table MSMC2 results and MSMC-IM estimates for all pairs of SGDP populations analyzed in this paper, *see separate Excel file*. The columns reported are described within a legend included in the Excel file.
(XLSX)

S1 Text Derivation of MSMC2 and MSMC-IM theory, *see separate PDF file*. (PDF)
