# Supplementary figures and images for: Tracking human population structure through time from whole genome sequences
Source: PLoS Genet. 2020 Mar 9;16(3):e1008552. doi: 10.1371/journal.pgen.1008552 (PMC7082067; doi:10.1371/journal.pgen.1008552)

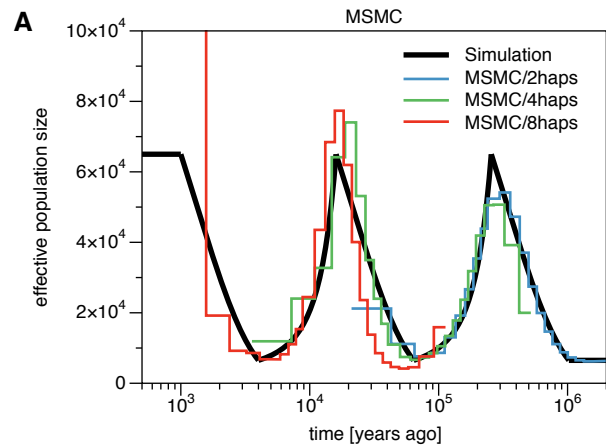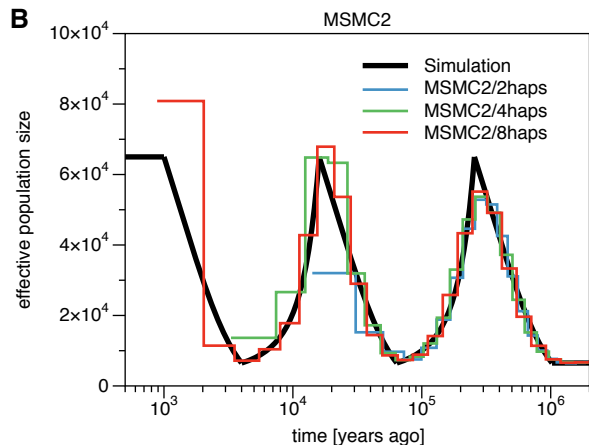

Supplement: S1 Fig — To test population size inference capabilities of MSMC (A) and MSMC2 (B) applied to two, four and eight haplotypes, we simulated a series of exponential population growths and declines, each changing the population size by a factor ten. The true population size is shown as dark solid line. Compared to MSMC, MSMC2 recovers the population size well, and the resolution in recent times increases with the number of haplotypes. With two haplotypes, MSMC2 infers the population history from 10kya to 3 million years, whereas, with four haplotypes and eight haplotypes the resolution in recent times is extended to 3kya and 1kya years ago respectively. (PDF) [file pgen.1008552.s001.pdf]

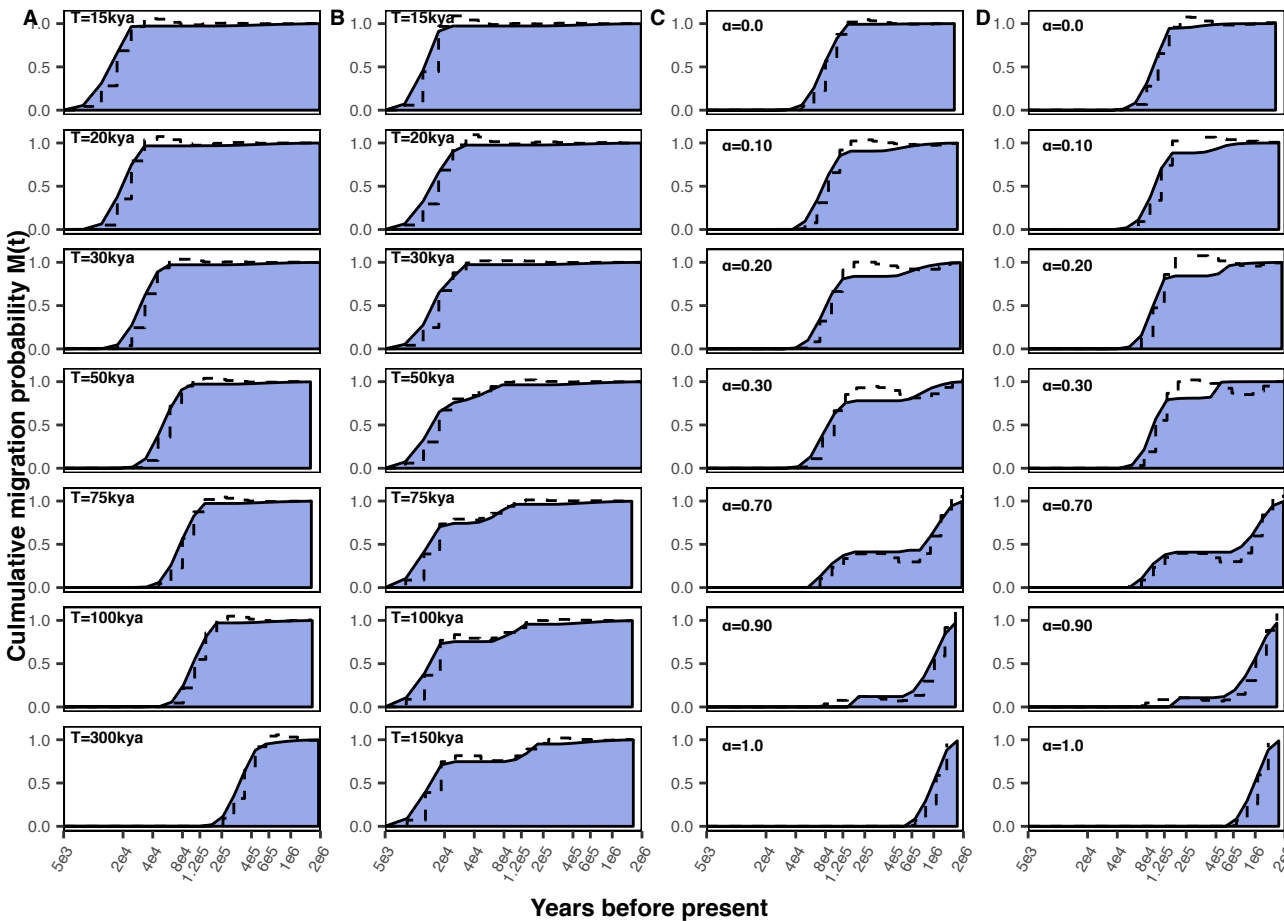

Supplement: S2 Fig — This figure shows the same results as Fig 2, but showing M(t) instead of m(t). The scenarios are (A) the Clean-split scenario. (B) the Split-with-migration scenario, and (C) the Split-with-archaic-admixture scenario. (D) the Split-with-archaic-admixture-and-bottleneck scenario. For panel (C) and (D), we show results with alpha ranging from 0 to 1, instead of between 0 to 20% shown in Fig 2. The relative CCR is shown in step-wise dashed lines to be compared with M(t). (PDF) [file pgen.1008552.s002.pdf]

A

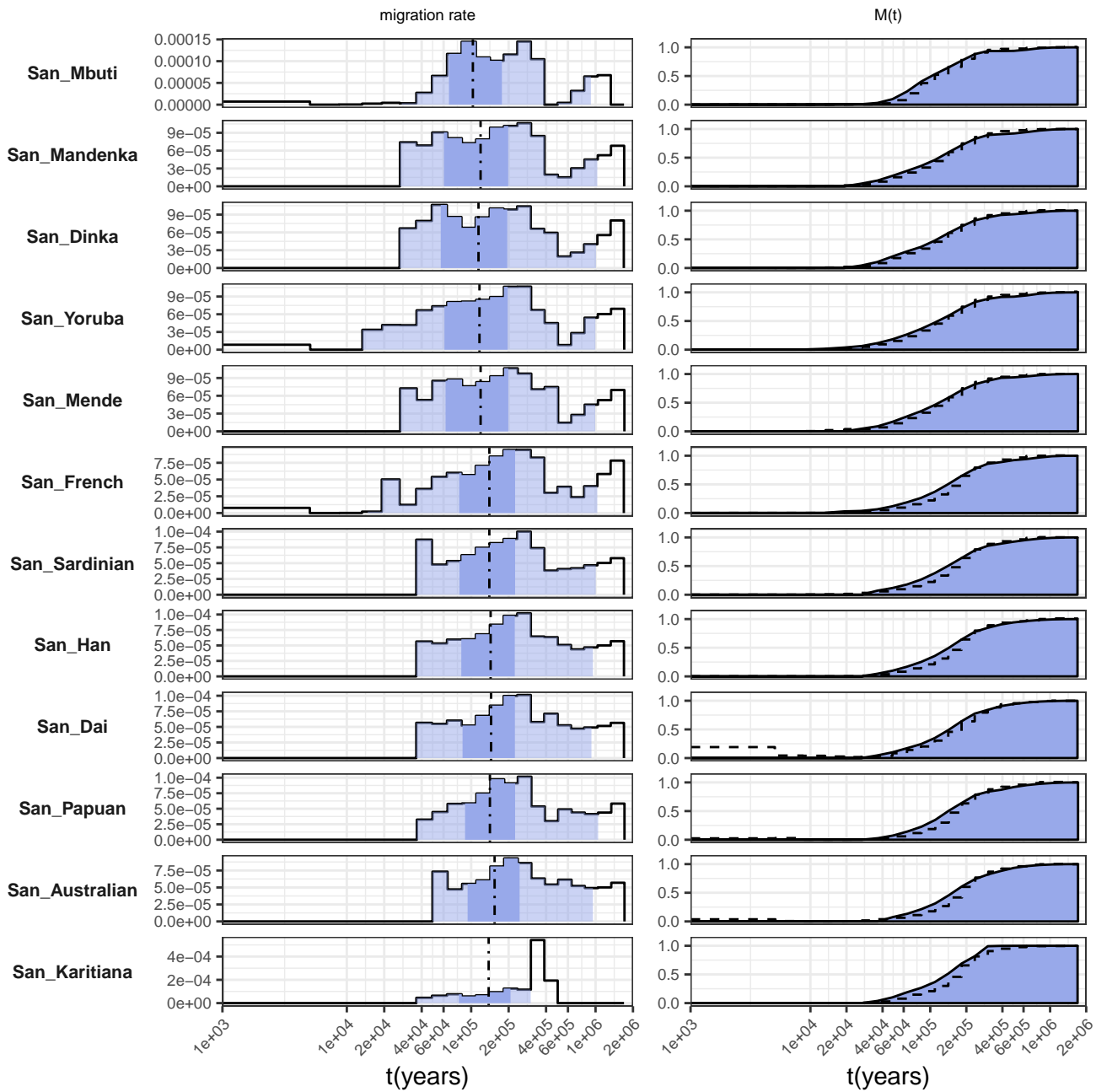

B

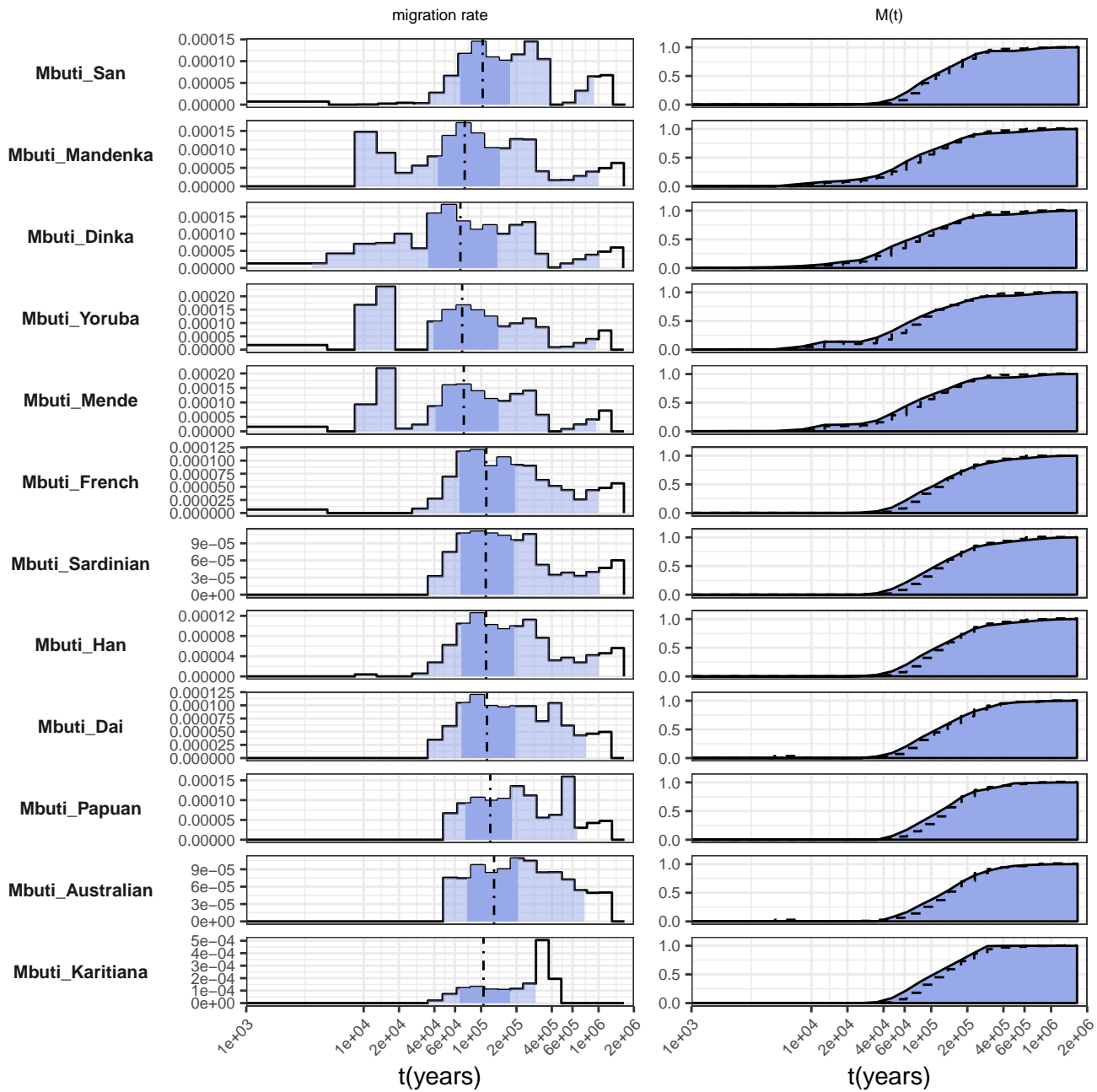

C

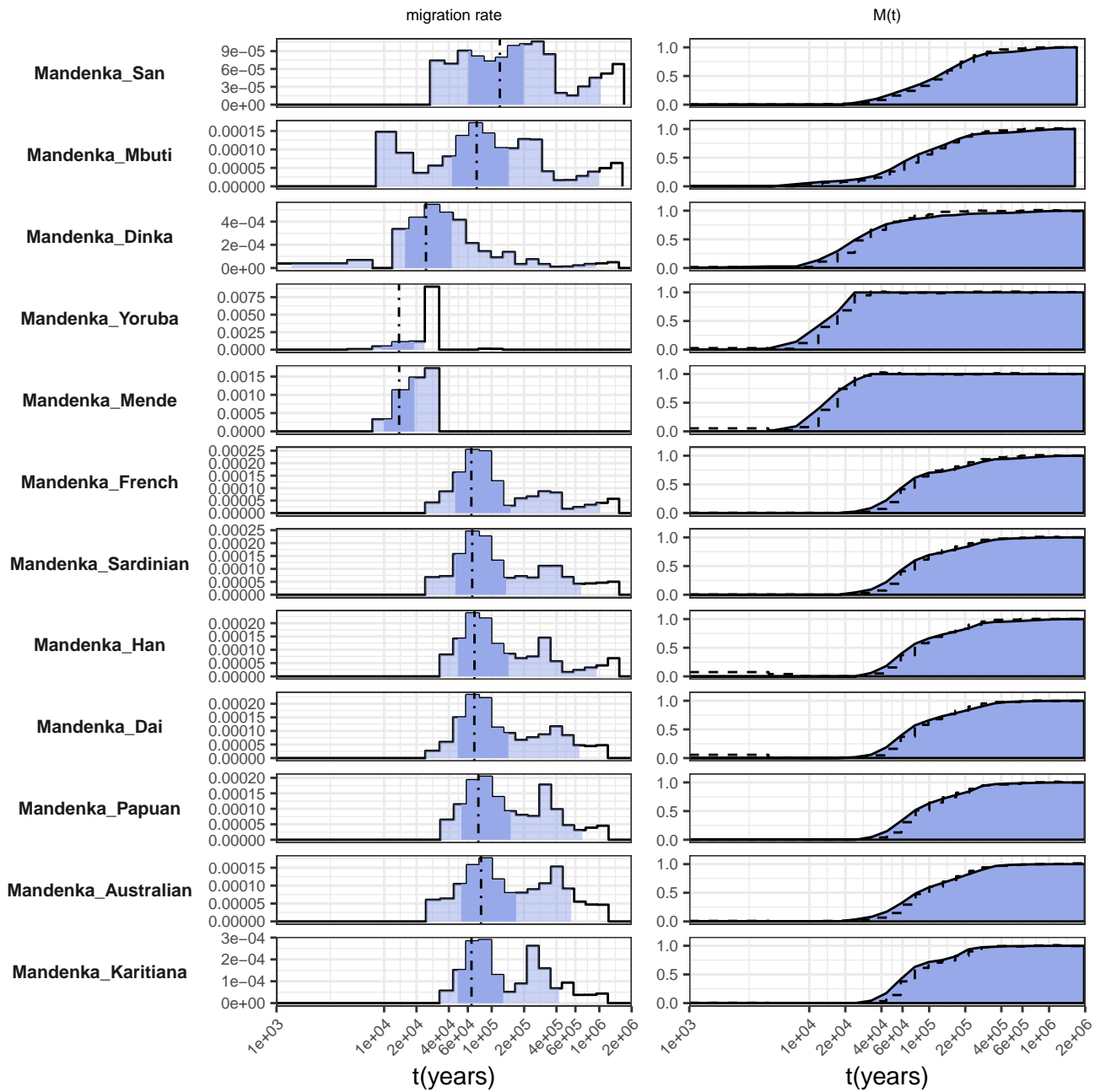

D

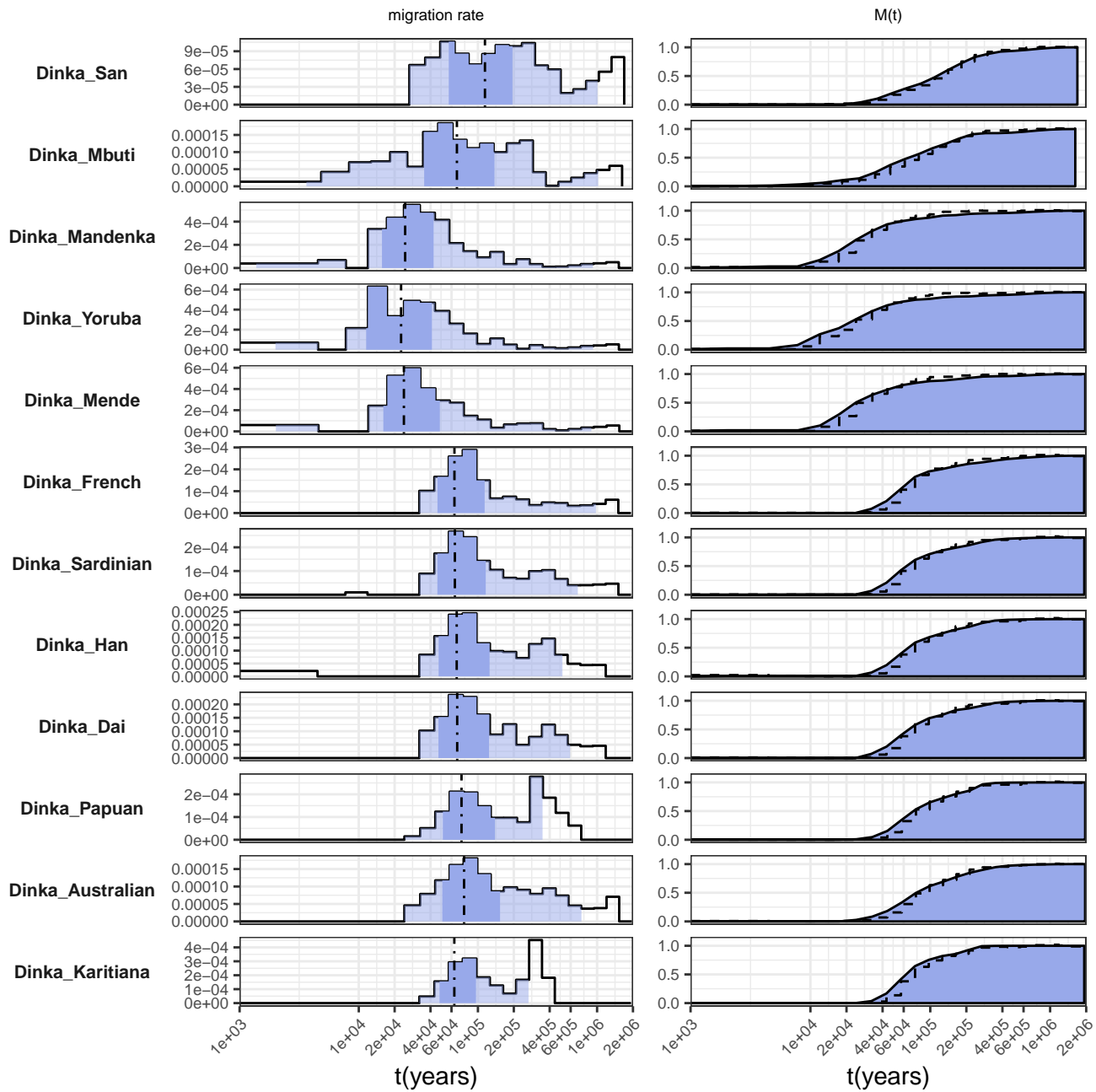

E

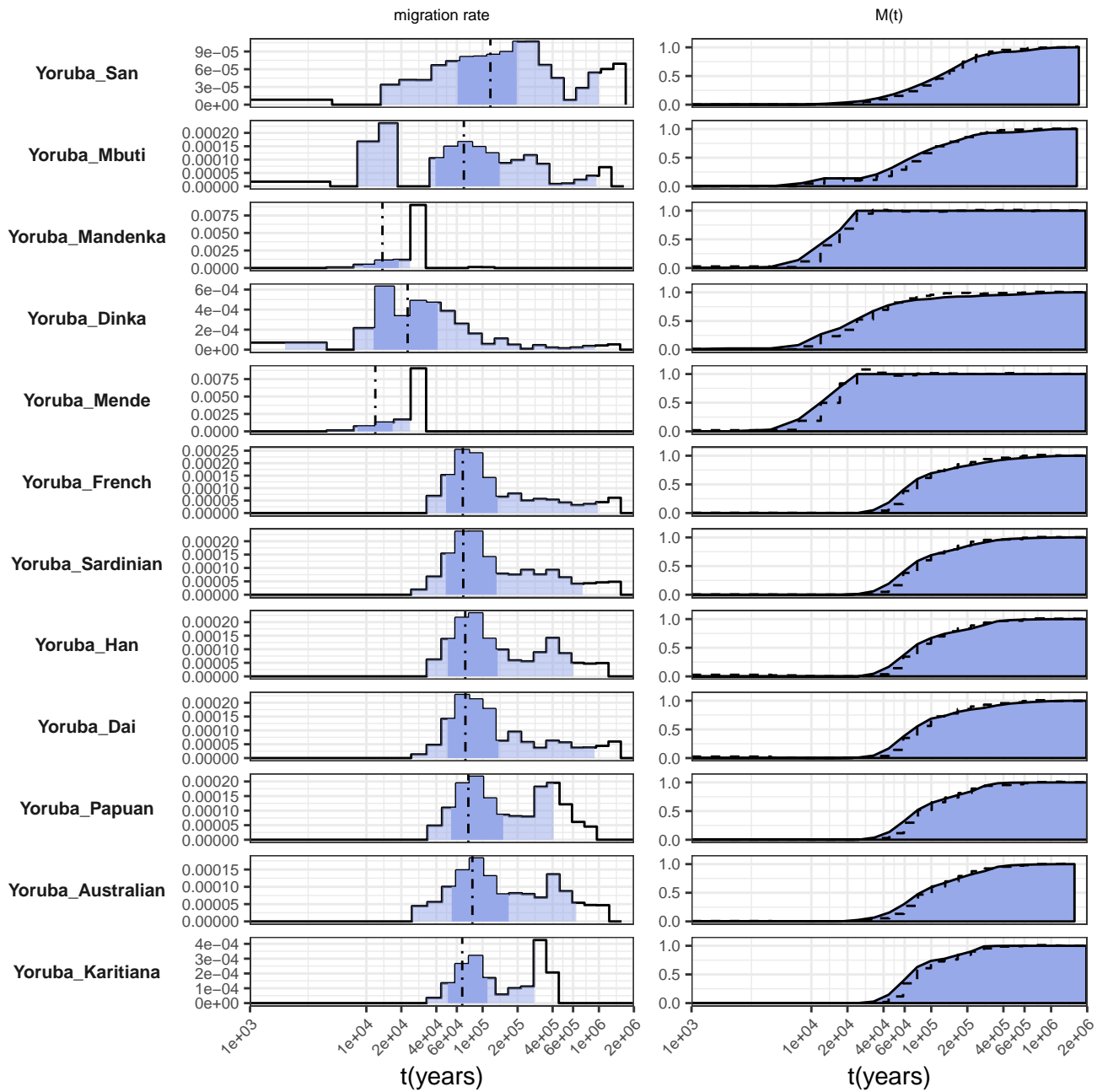

F

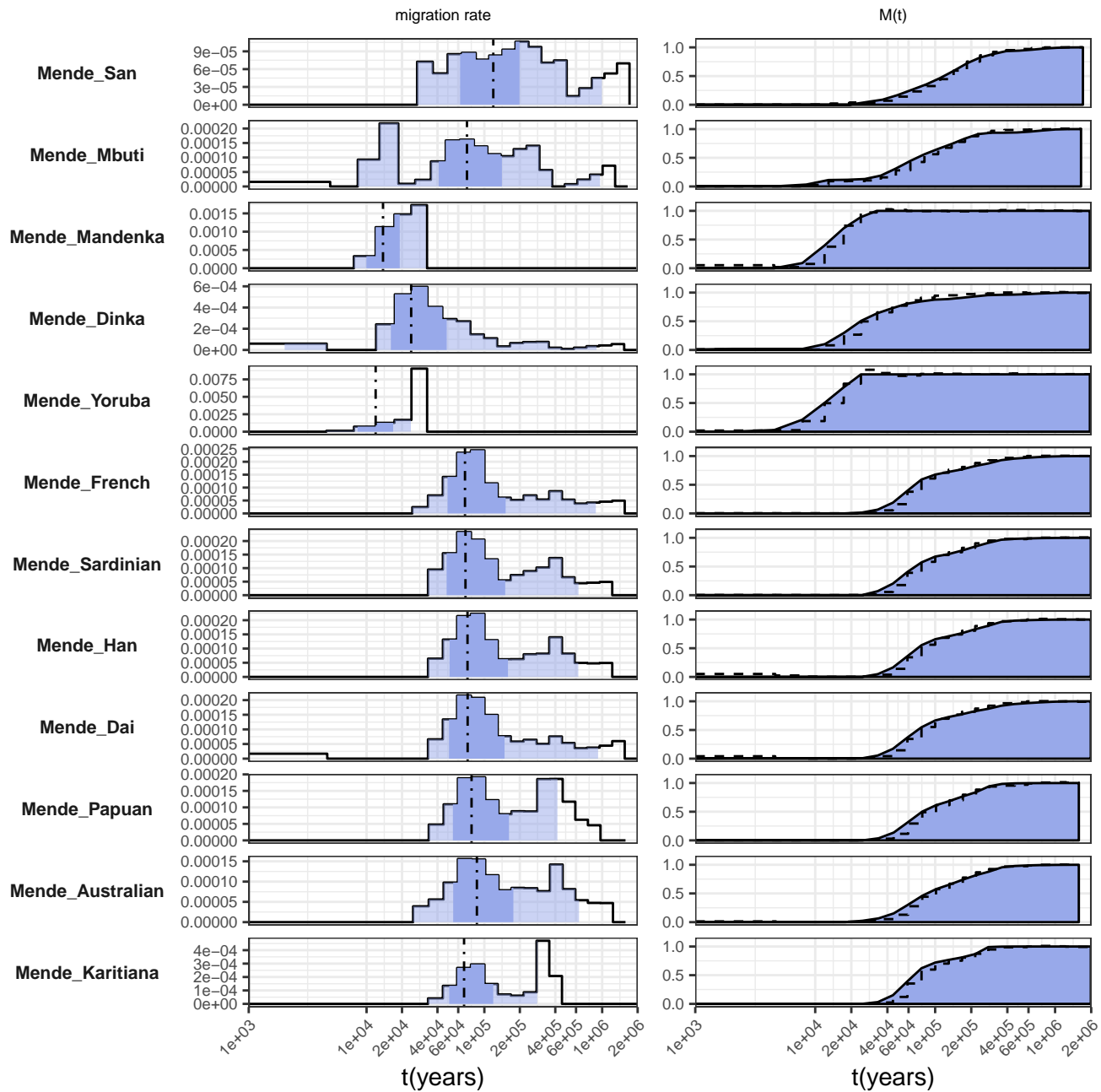

G

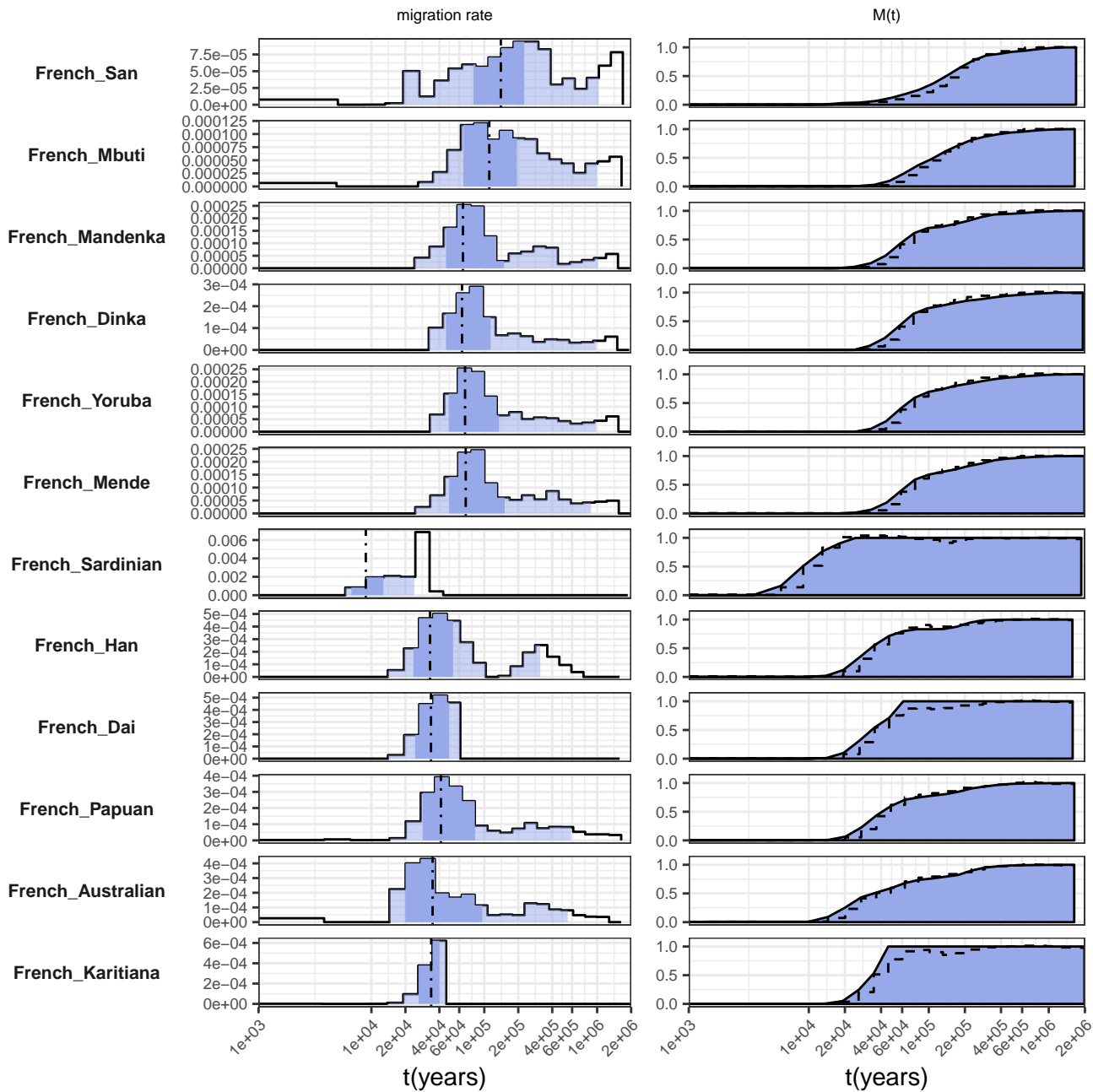

H

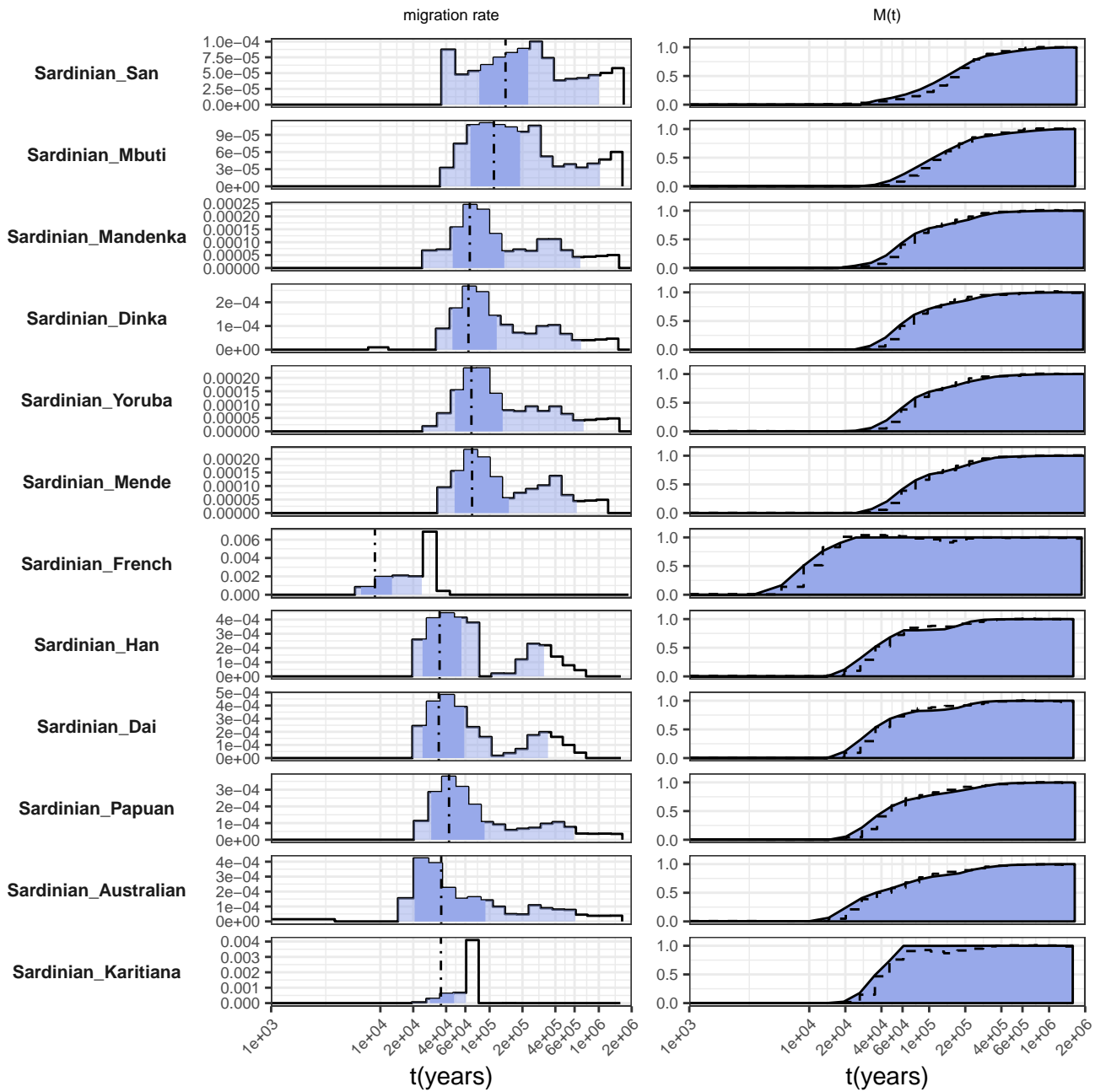

I

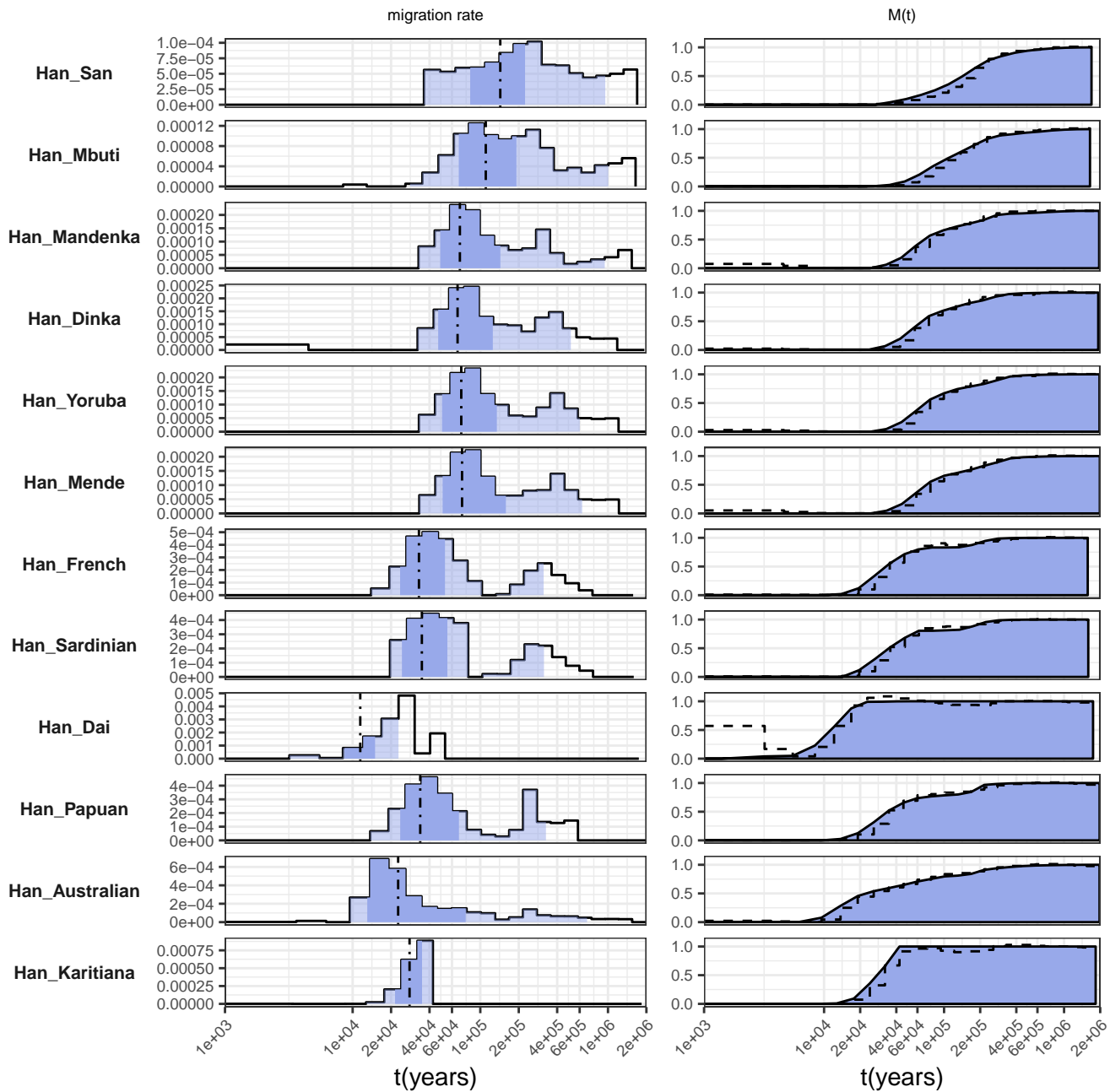

J

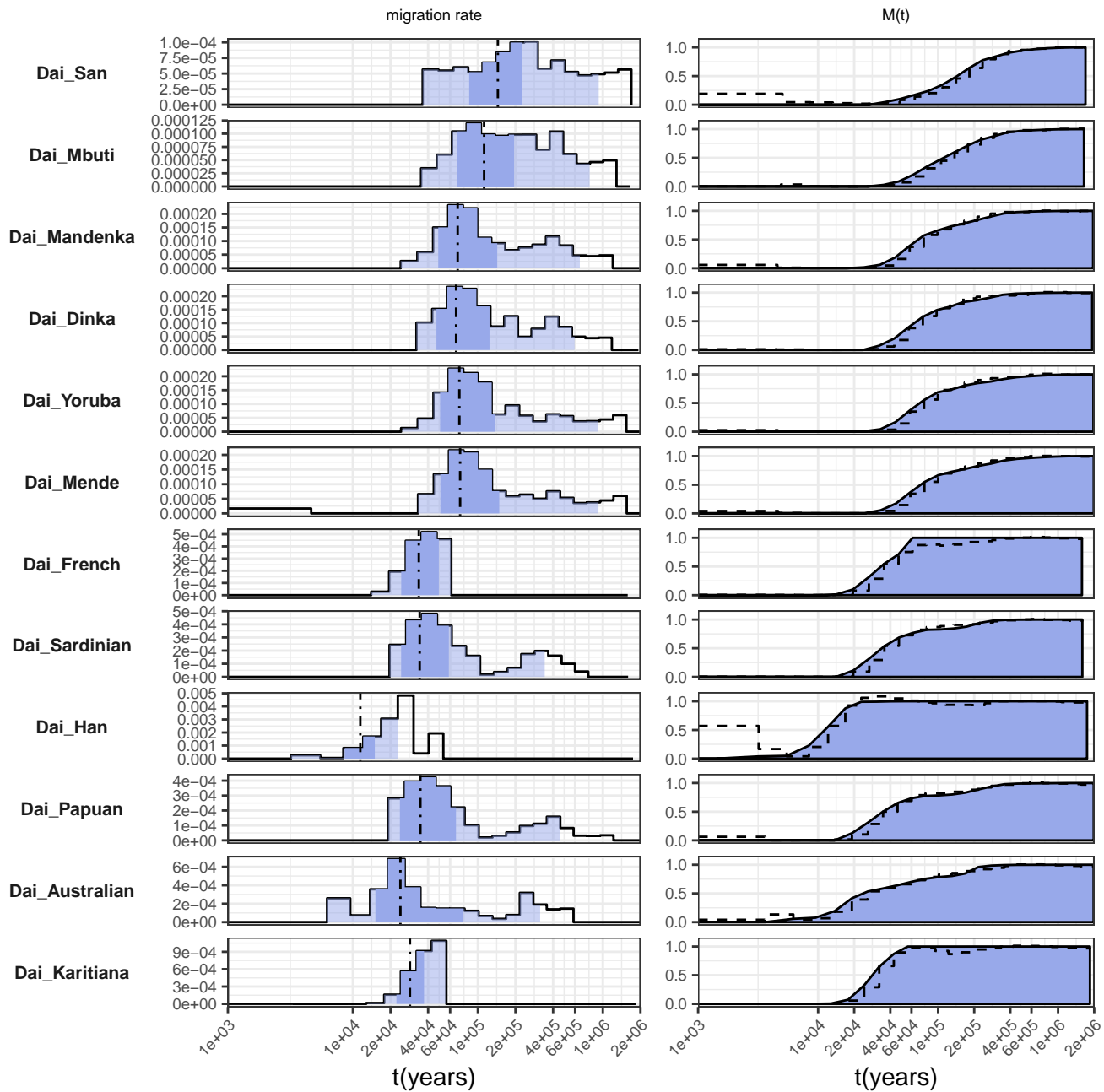

K

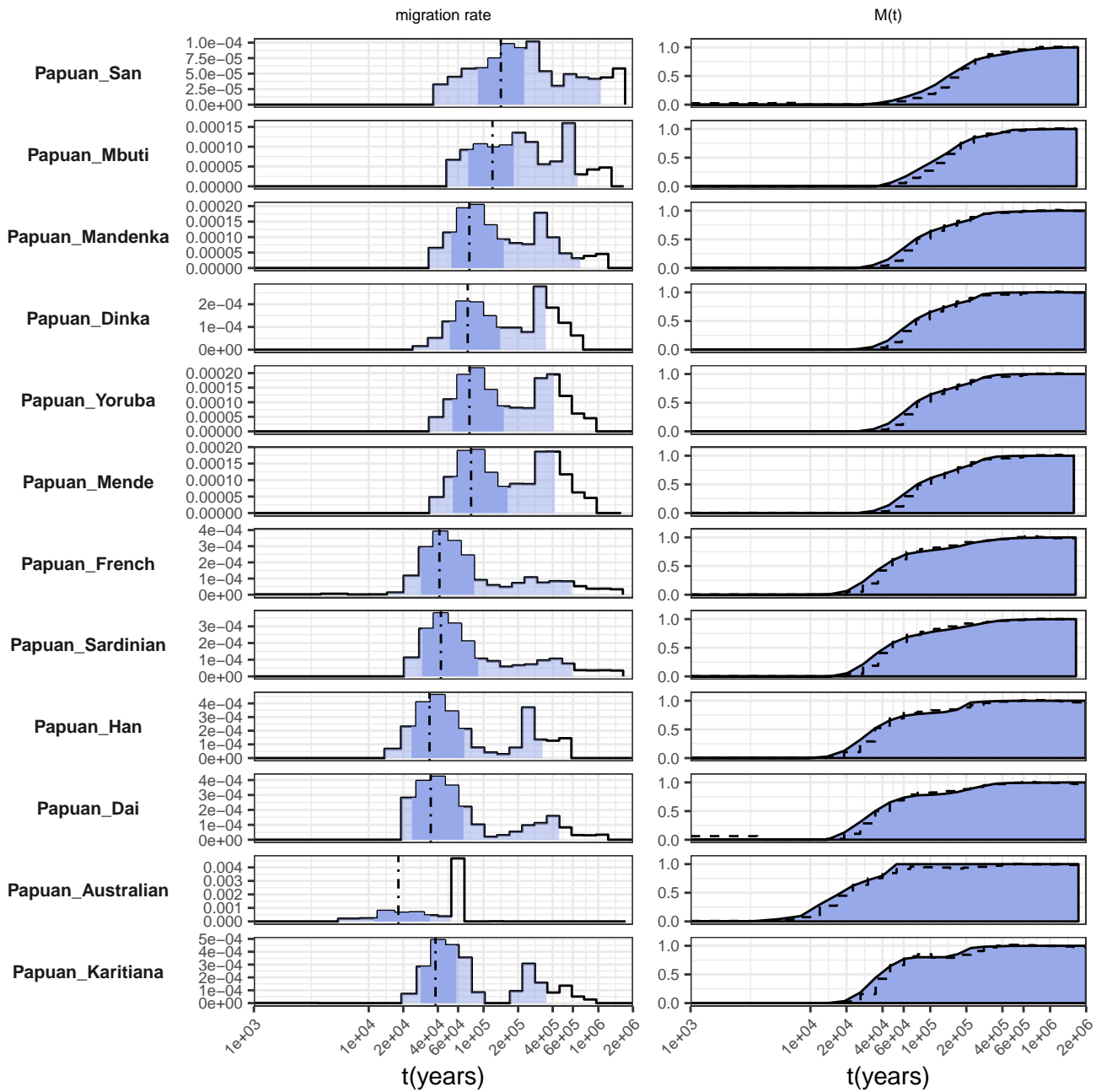

L

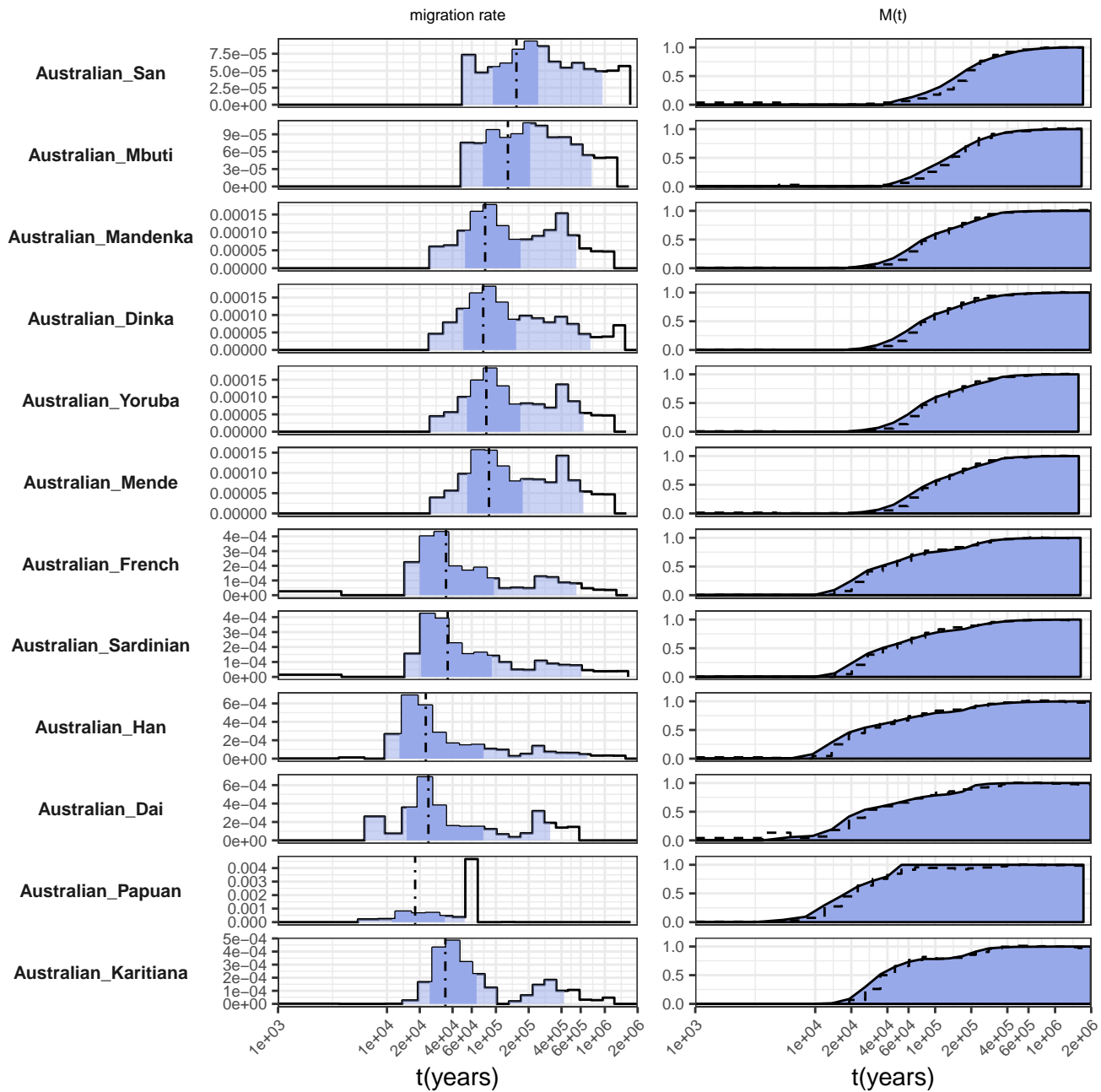

M

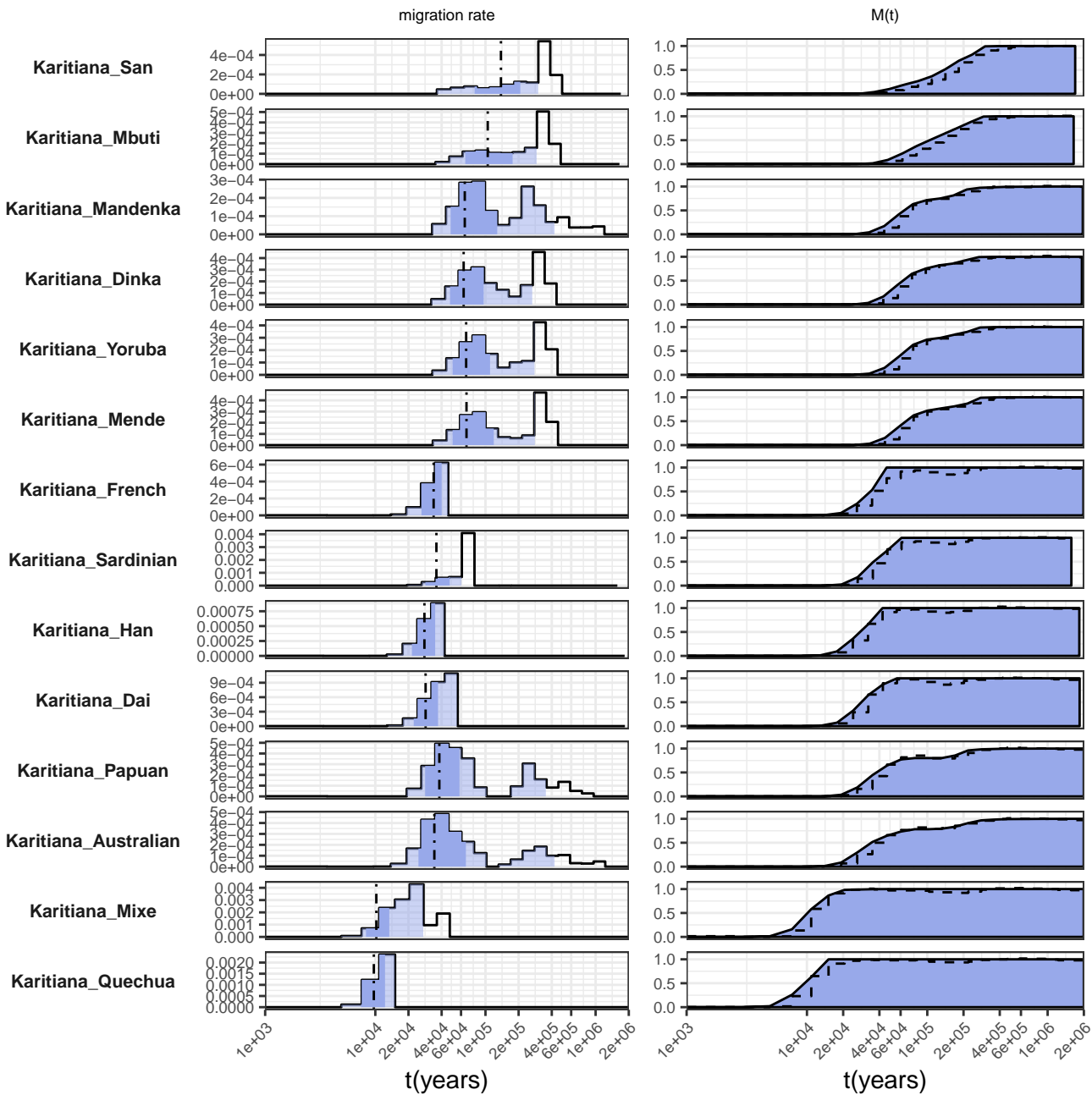

Supplement: S4 Fig — Pairwise migration profiles for 13 worldwide populations, involving San (A), Mbuti (B), Mandenka (C), Dinka (D), Yoruba (E), Mende (F), French (G), Sardinian (H), Han (I), Dai (J), Papuan (K), Australian (L), Karitiana (M). The relative CCR is shown in step-wise dashed lines to be compared with M(t). See separate joint PDF file. (PDF) [file pgen.1008552.s004.pdf]

A

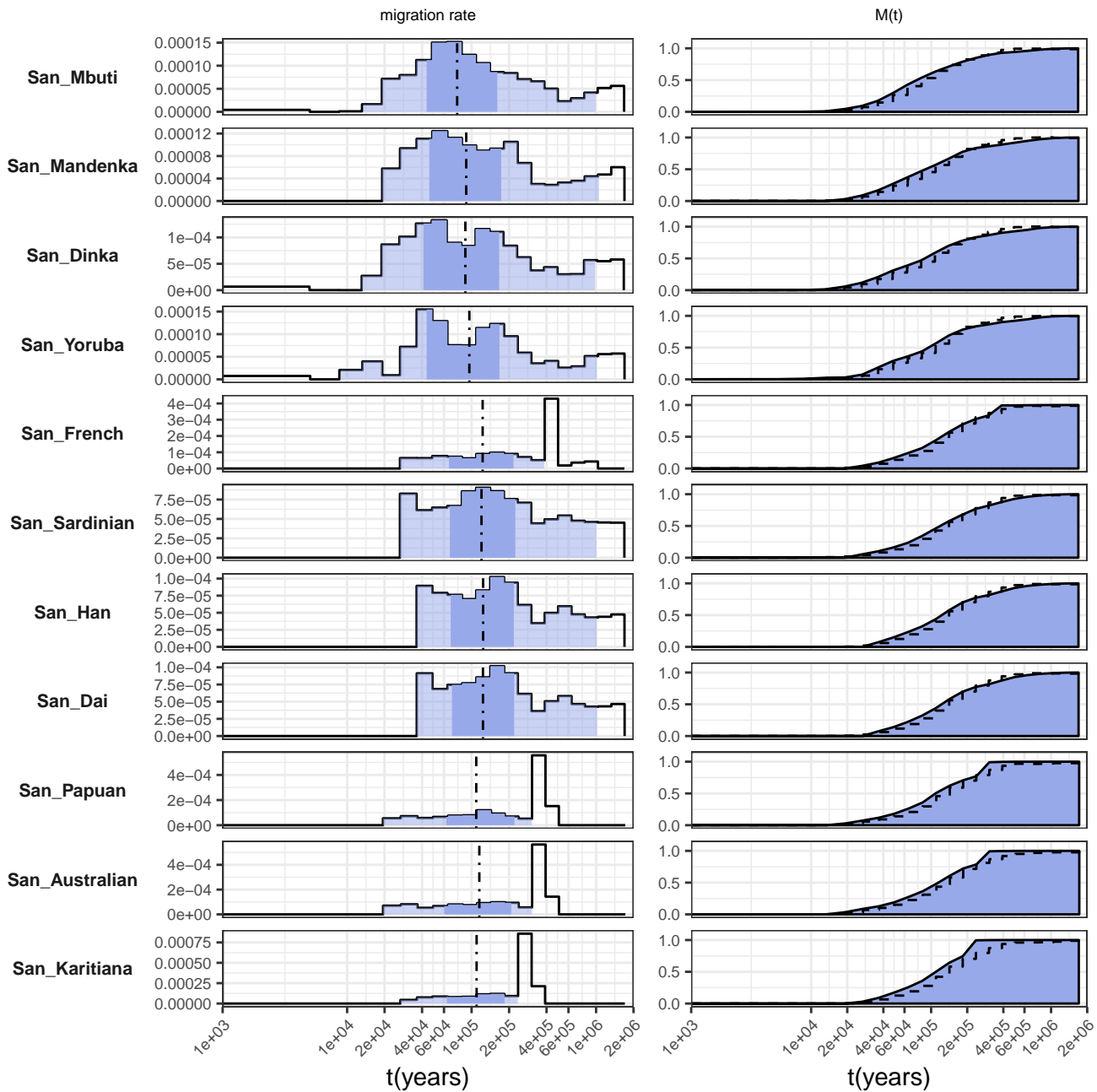

B

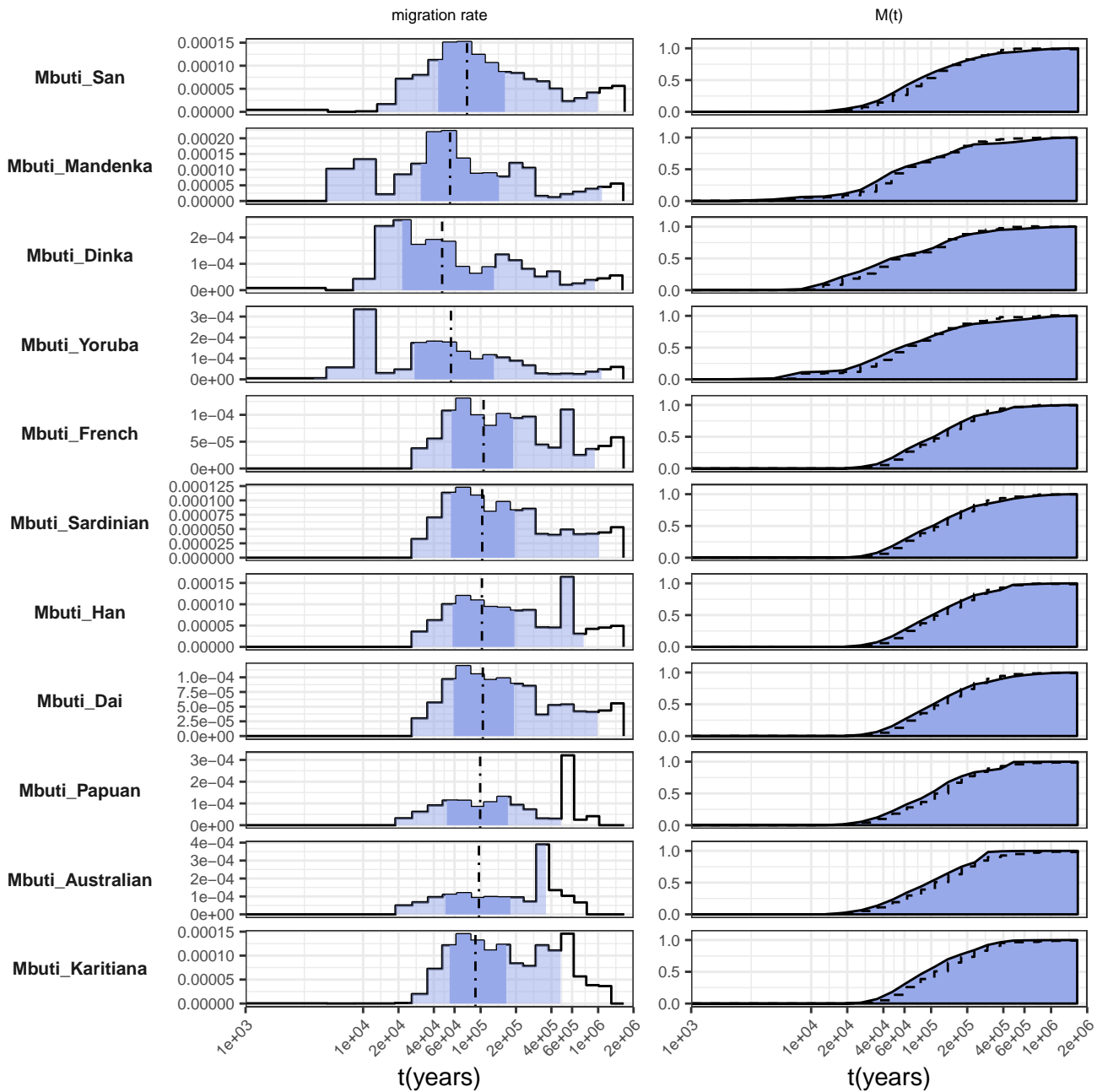

C

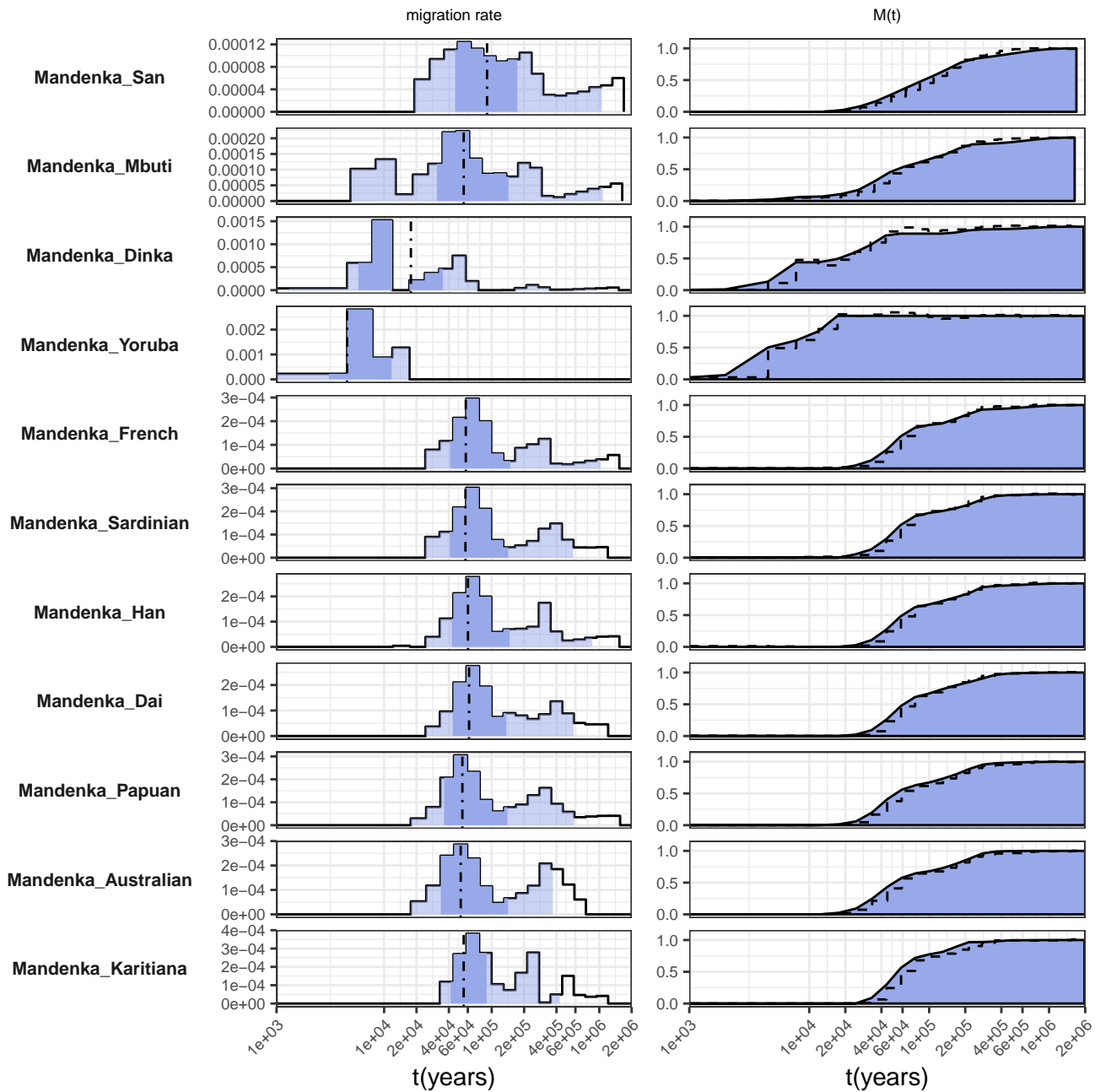

D

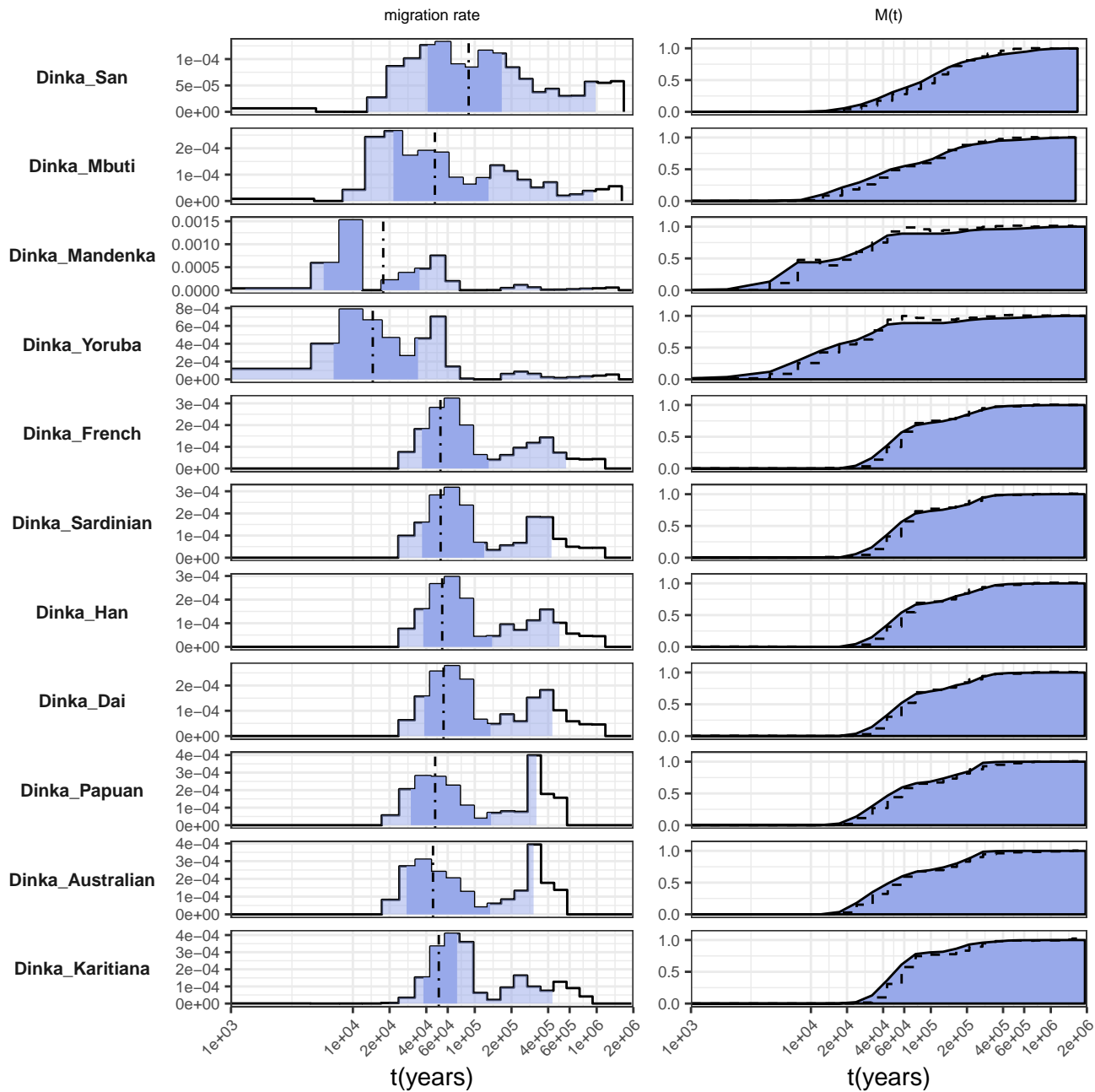

E

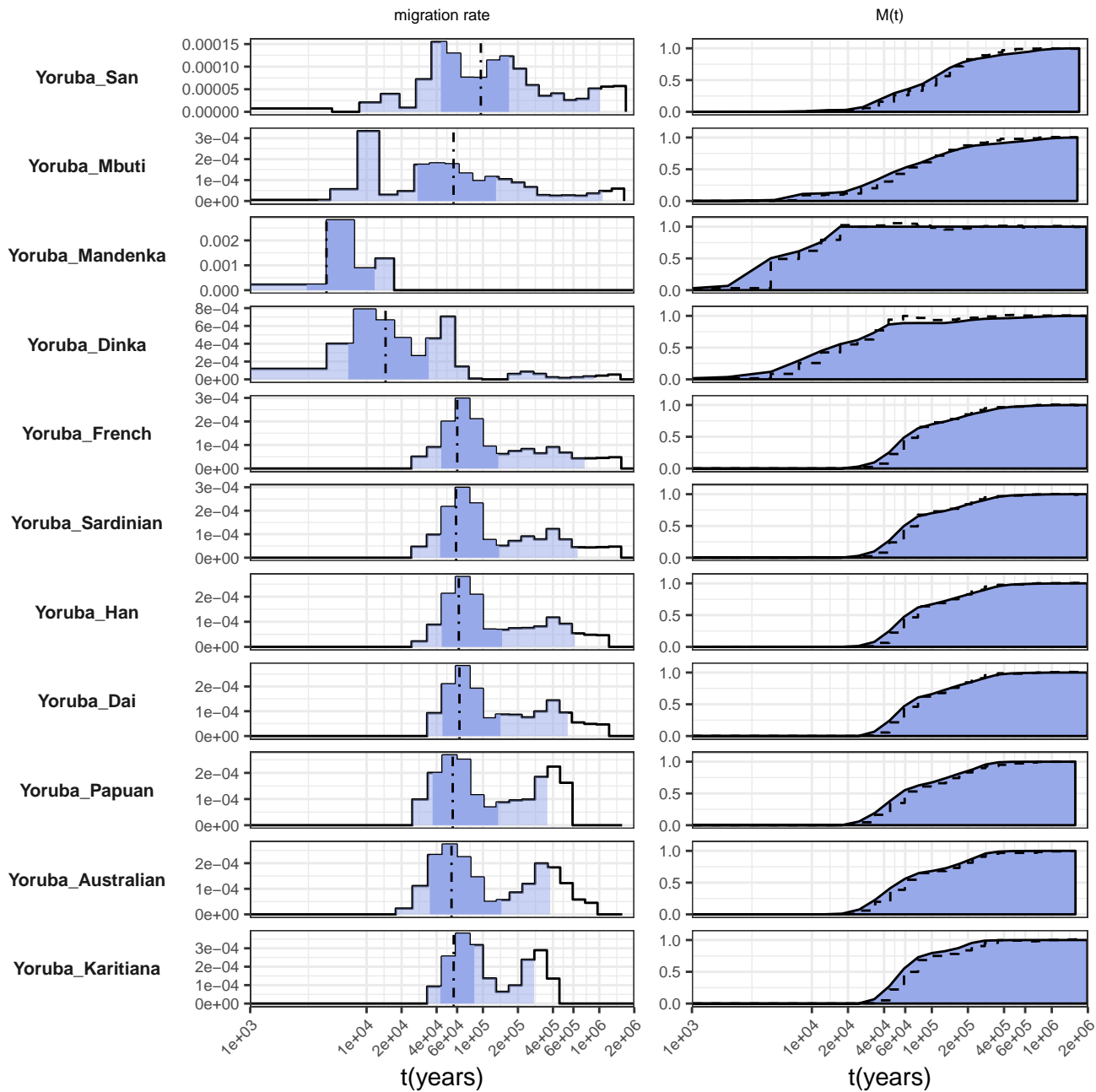

F

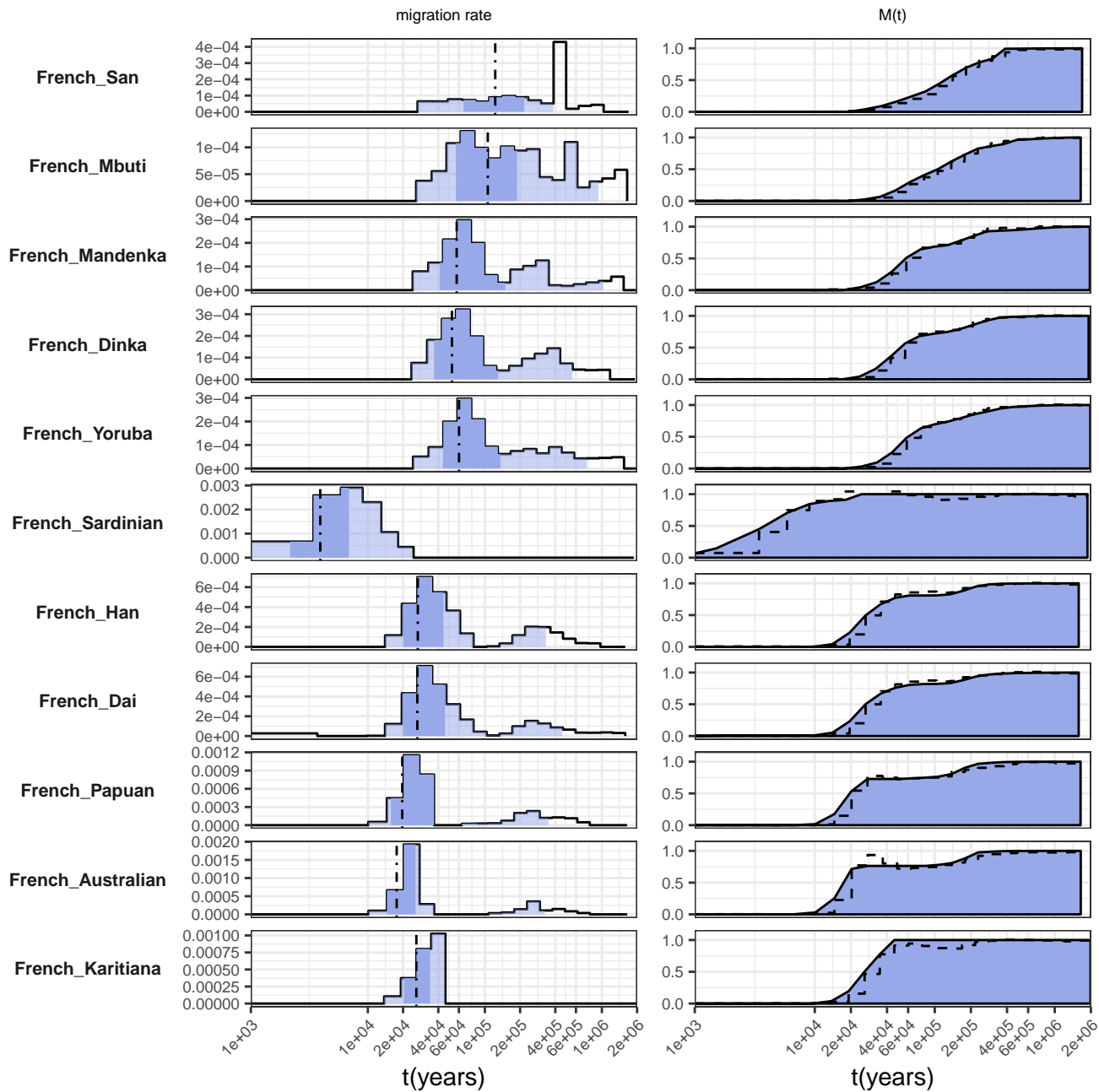

G

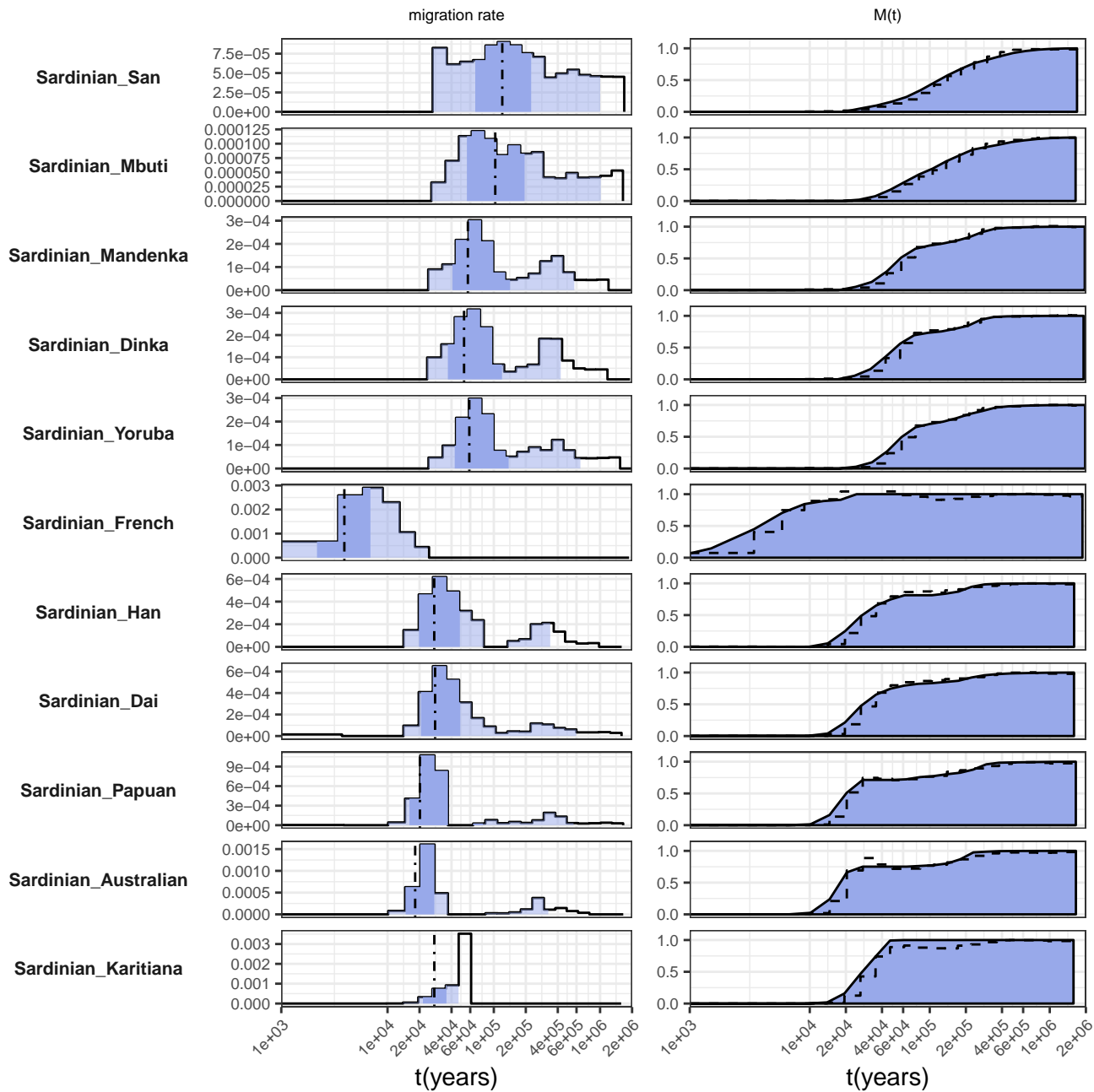

H

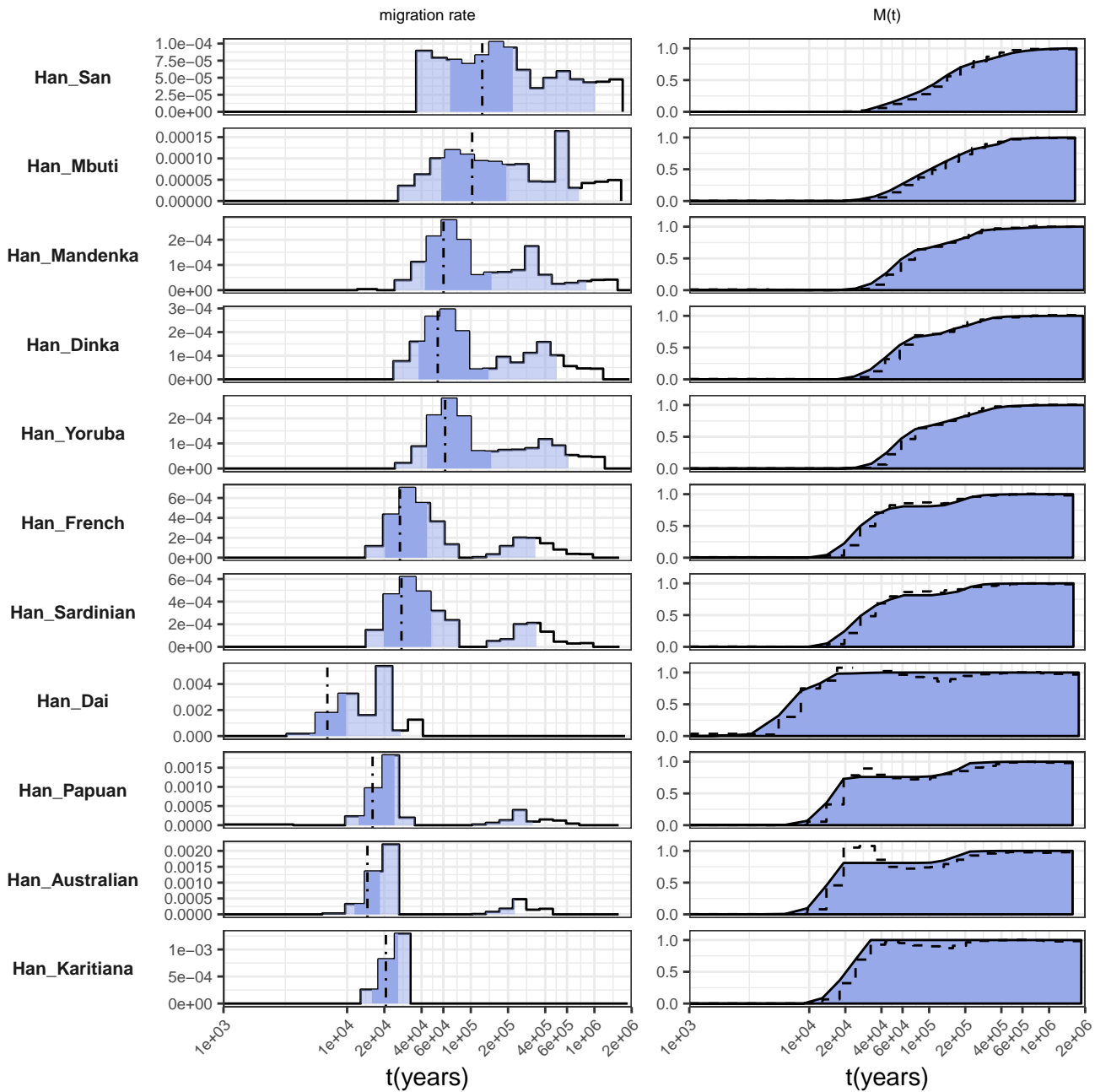

1

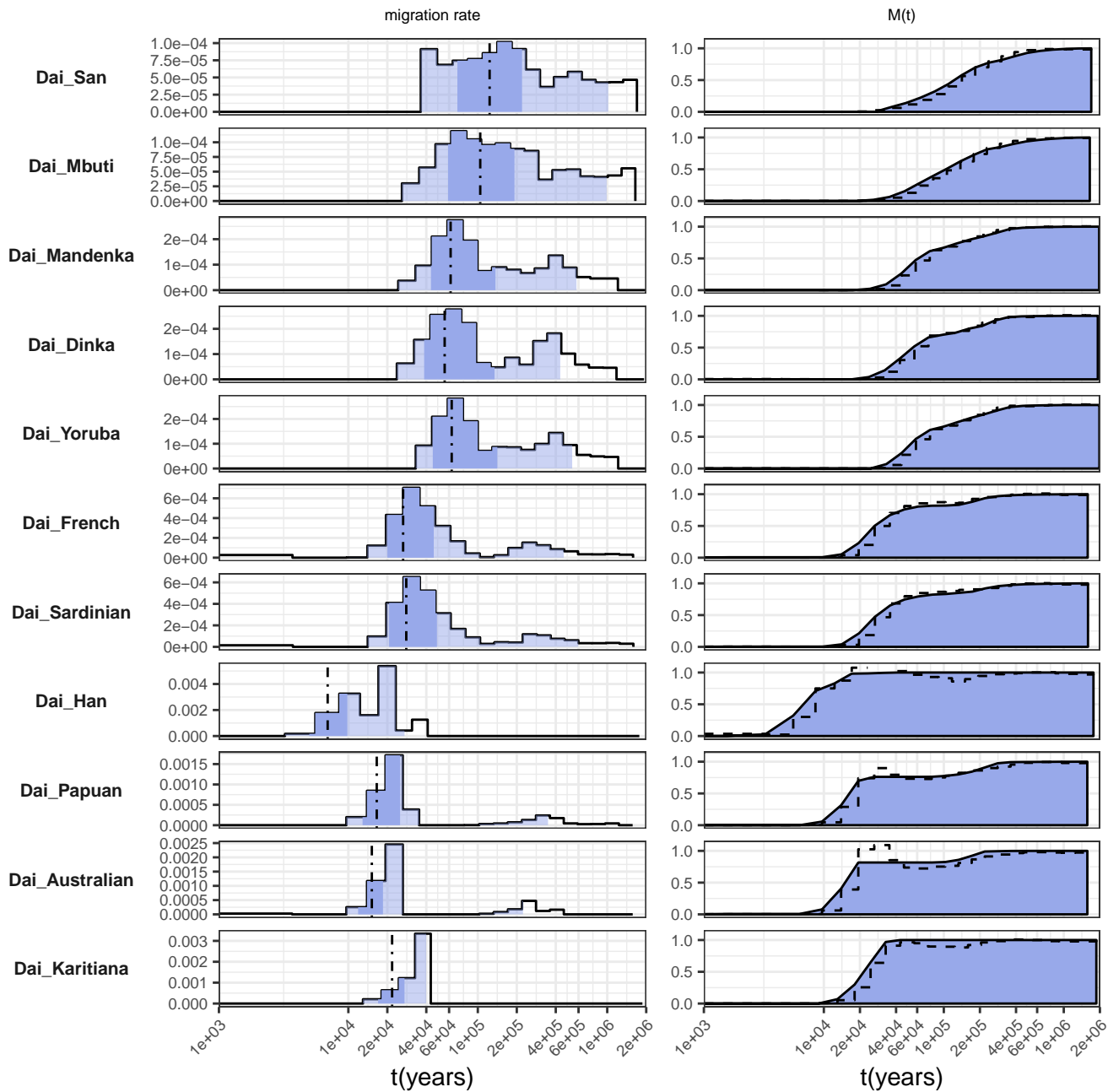

J

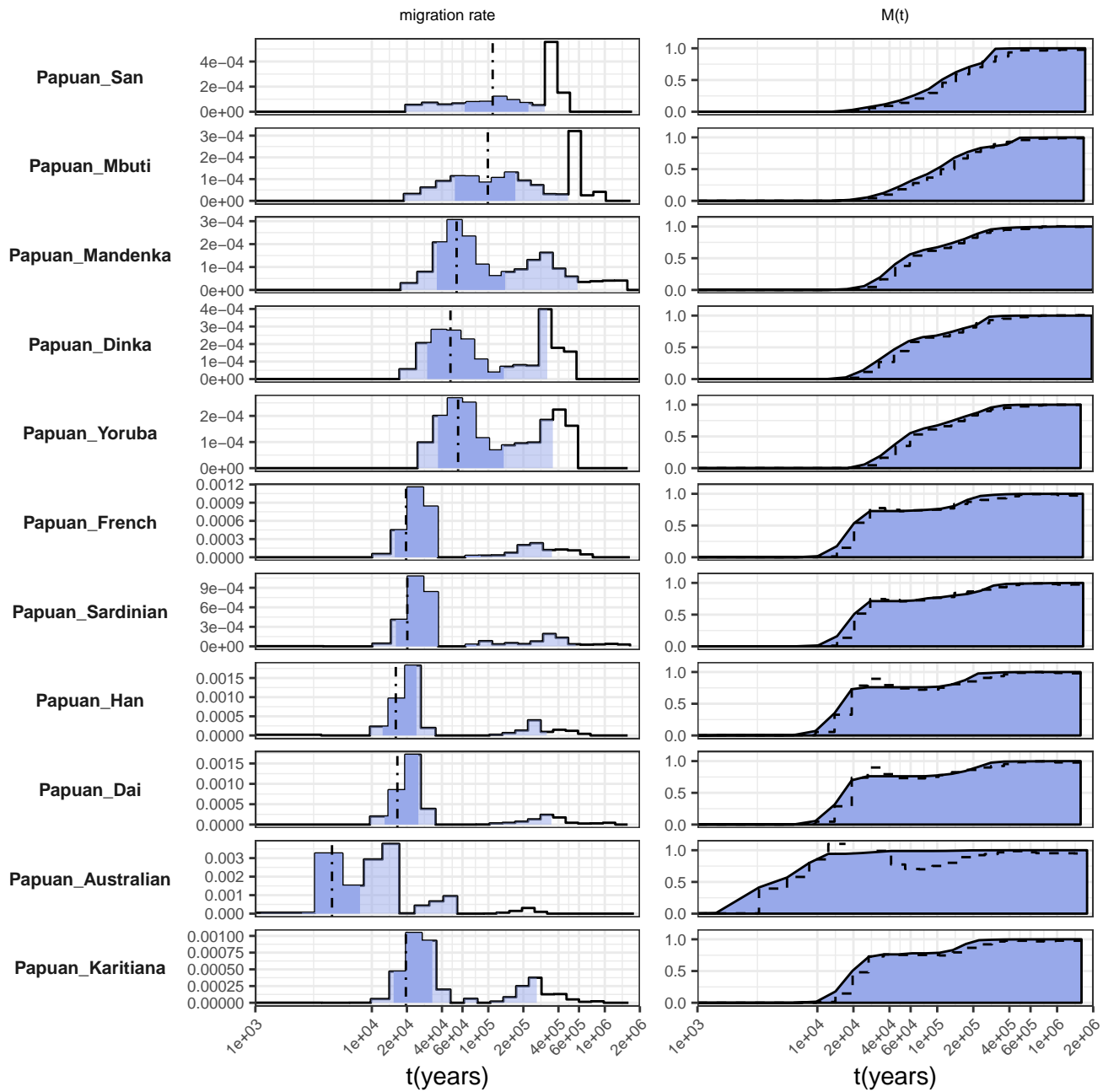

K

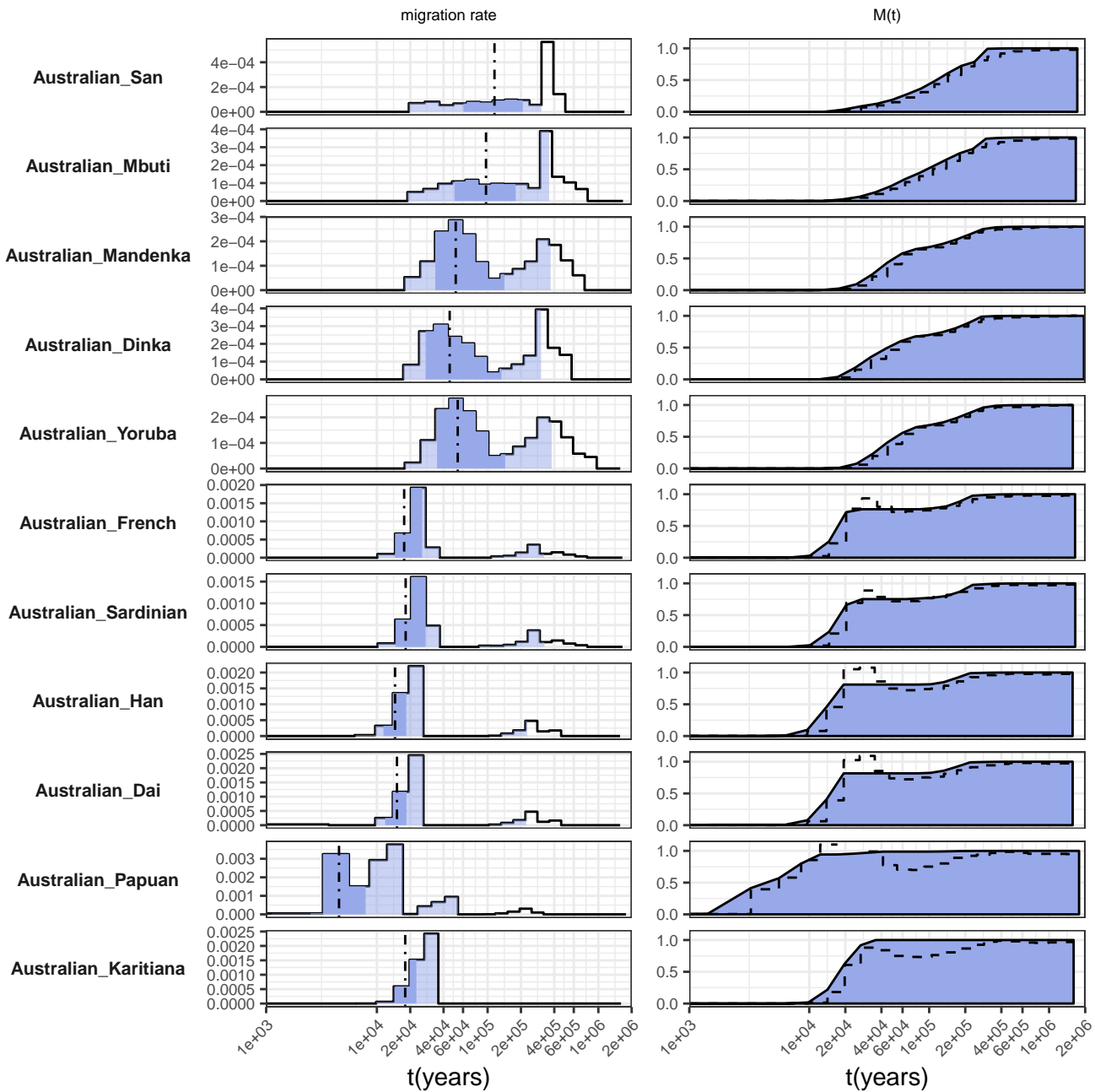

L

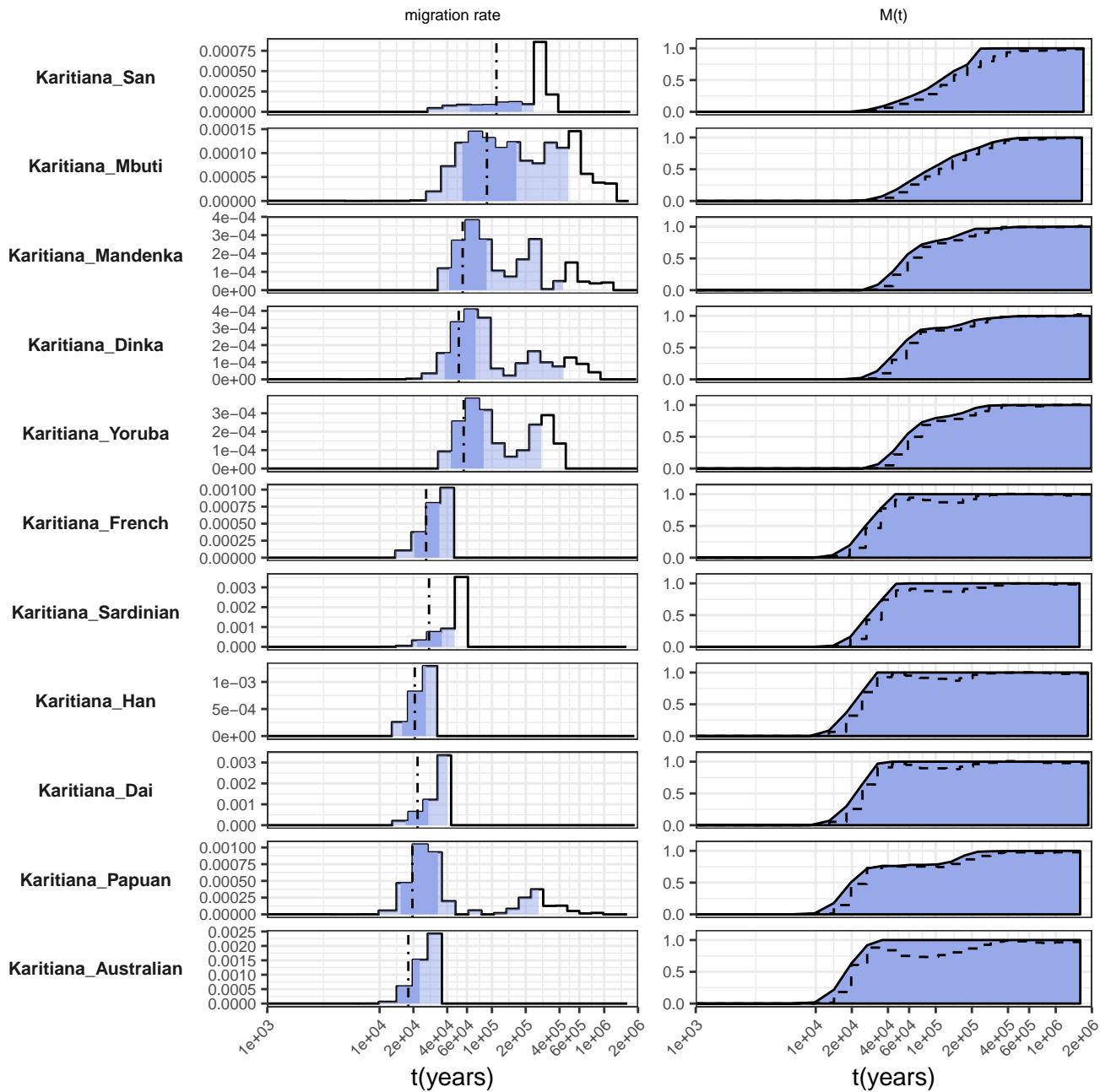

Supplement: S5 Fig — Here we have analyzed 12 worldwide populations from Prüfer et al (2014) with independent data processing as described in Methods: San (A), Mbuti (B), Mandenka (C), Dinka (D), Yoruba (E), French (F), Sardinian (G), Han (H), Dai (I), Papuan (J), Australian (K), Karitiana (L). The relative CCR is shown in step-wise dashed lines to be compared with M(t). See separate joint PDF file. (PDF) [file pgen.1008552.s005.pdf]

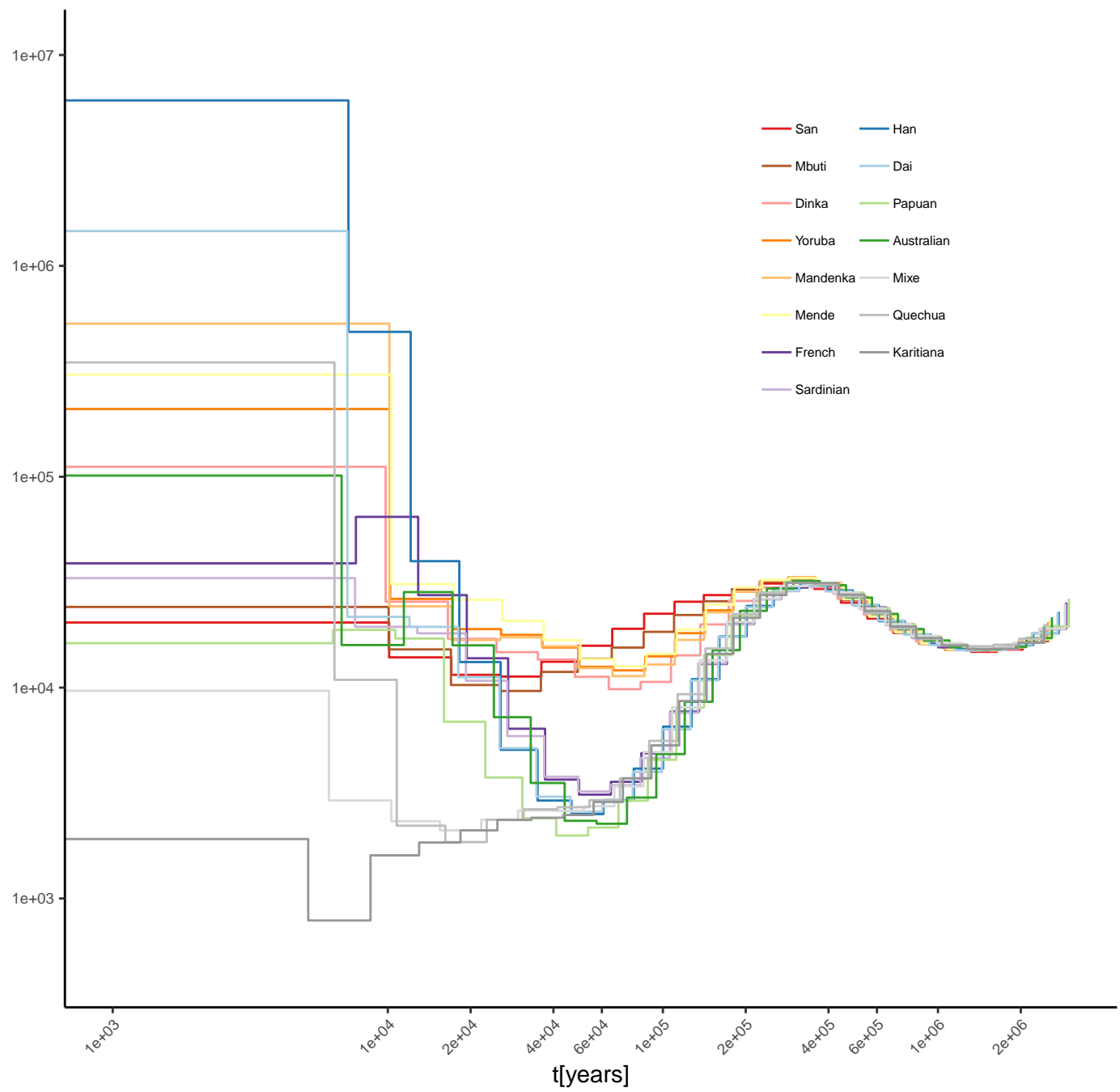

Supplement: S6 Fig — We show the estimates from MSMC using 8 haplotypes/4 individuals per population from the SGDP dataset. (PDF) [file pgen.1008552.s006.pdf]

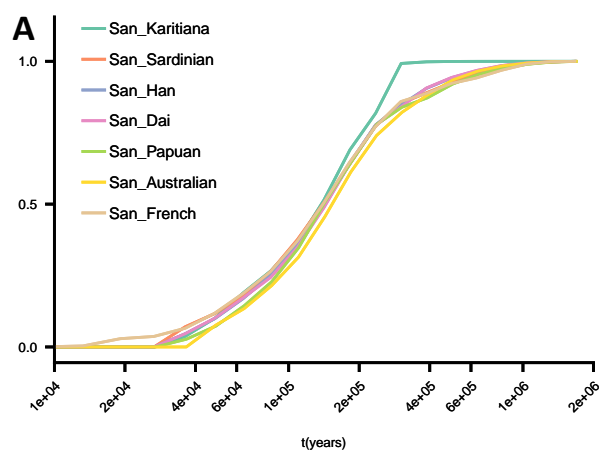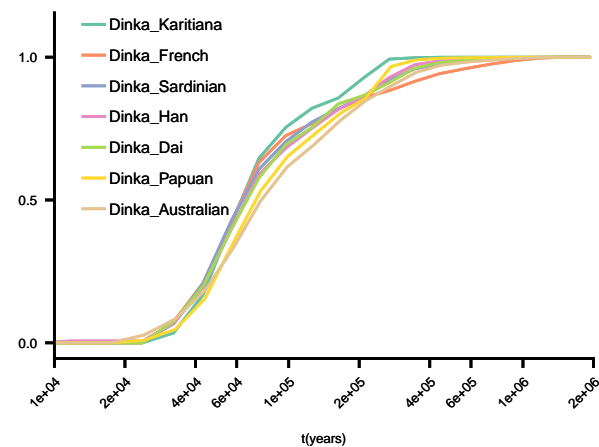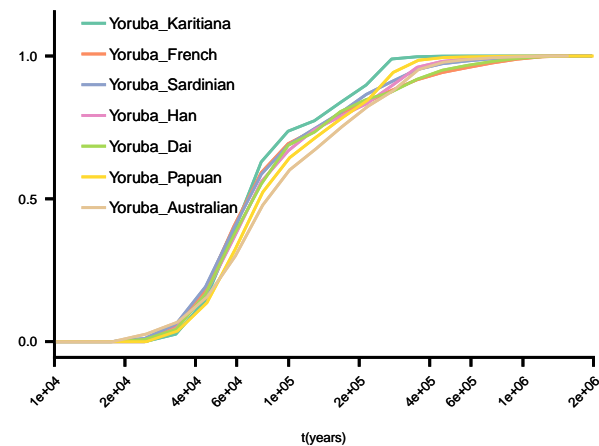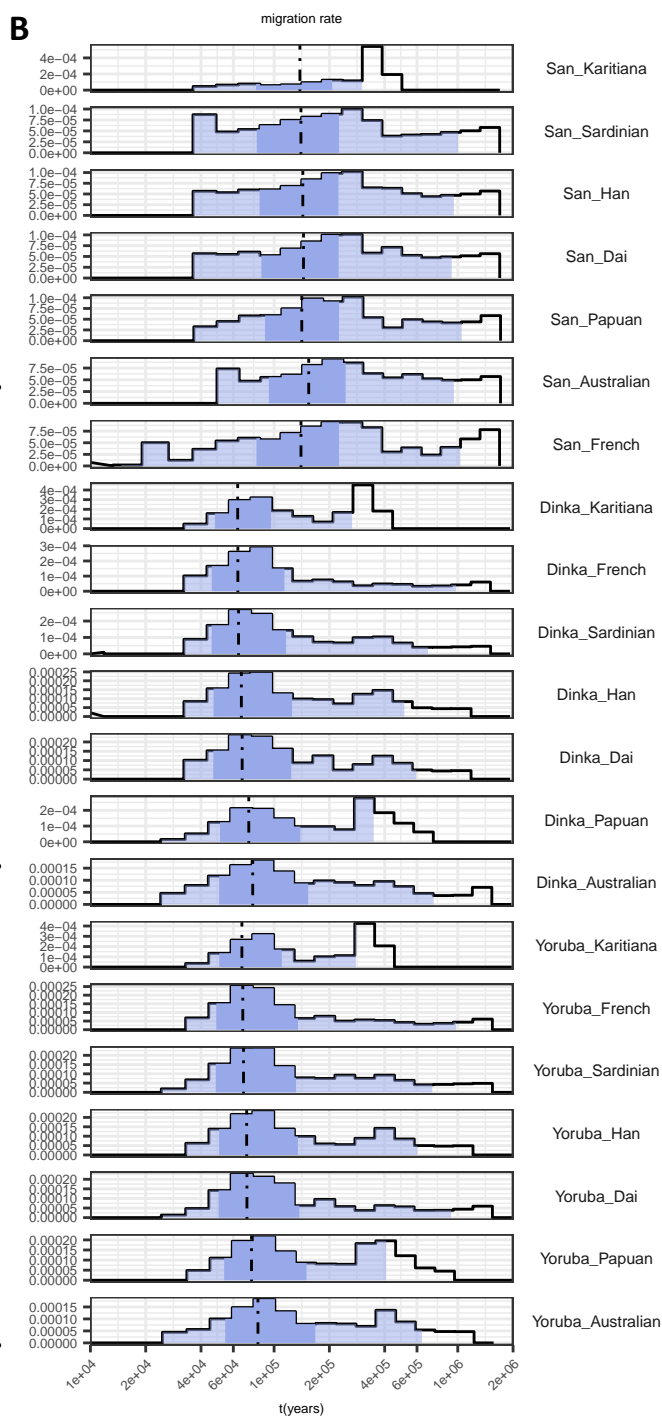

Supplement: S7 Fig — Here we show analyses on the divergence of Papuans and Australians from Africans vs. other Non-African populations from Africans. We show the cumulative migration probability M(t) in (A), and the migration rate m(t) (B) for pairs of populations of Yoruba, Dinka and San with one non-African population as indicated. (PDF) [file pgen.1008552.s007.pdf]

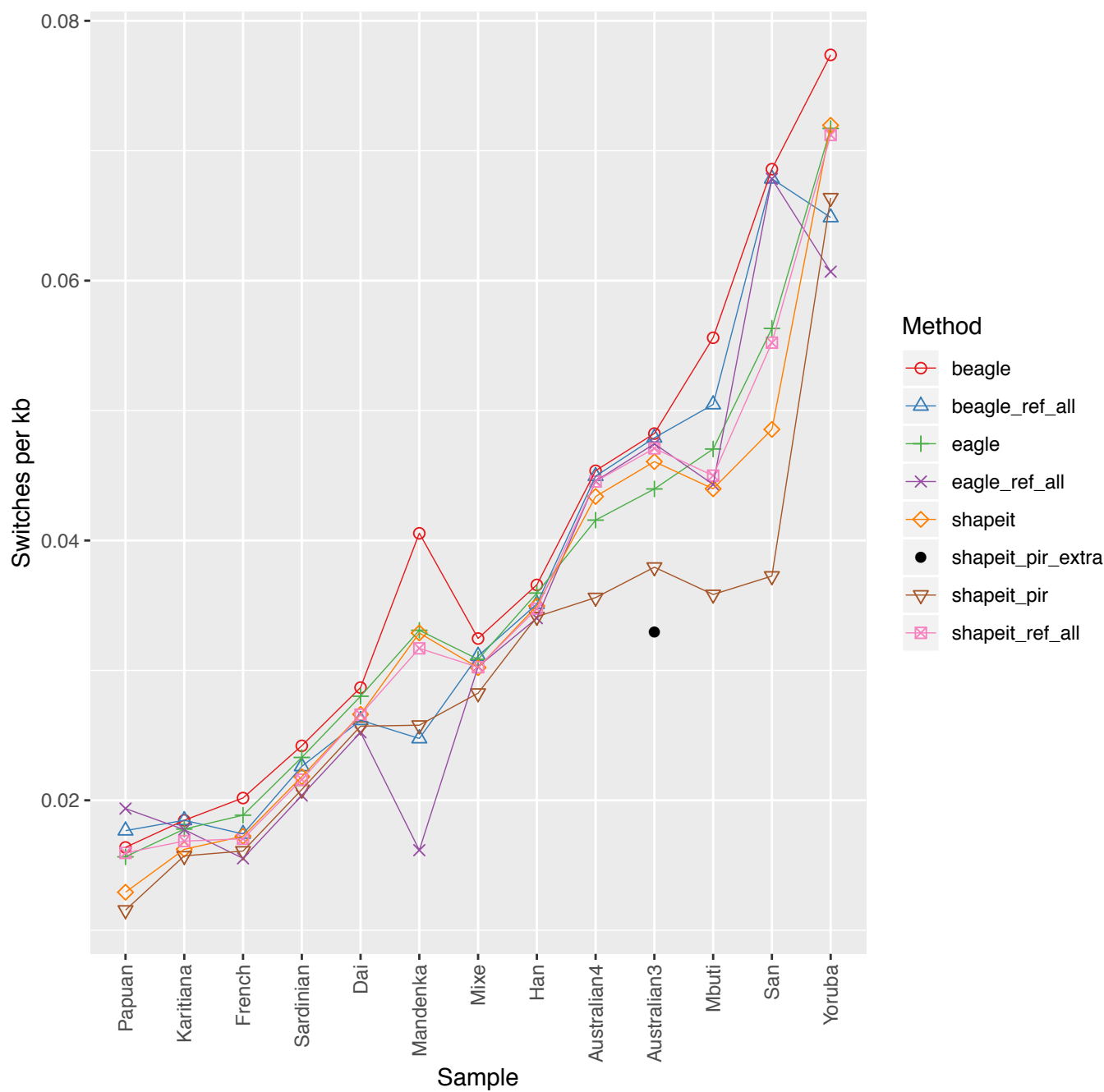

Supplement: S8 Fig — beagle and beagle_ref_all denote BEAGLE phasing without and with reference panel (here and below denoting the 1000 Genomes Phase 3 reference panel). eagle and eagle_ref_all represent EAGLE phasing without and with reference panel. shapeit and shapeit_ref_all represent SHAPEIT phasing without and with reference panel. shapeit_pir represents SHAPEIT phasing with phase-informative reads. shapeit_pir_extra represents SHAPEIT phasing with long-insert-size reads as additional phase informative reads, which was applied to B-Australian-3 only. See Methods for details. (PDF) [file pgen.1008552.s008.pdf]

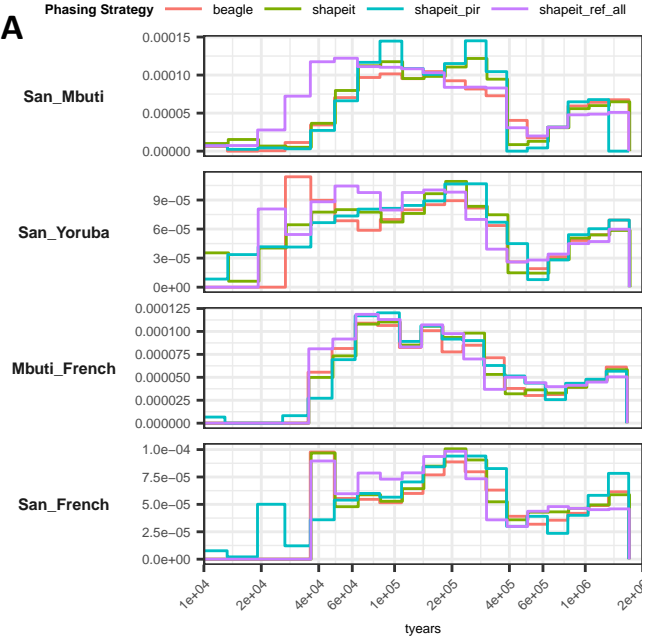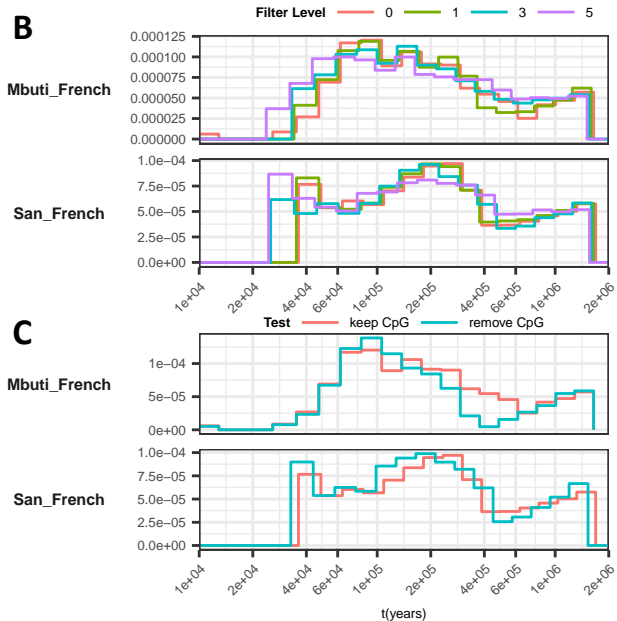

Supplement: S9 Fig — We show (A) the impact of the phasing strategy using San/Mbuti, San/Yoruba, Mbuti/French and San/French as examples, (B) the impact of the filtering level for generating individual masks using San/French and Mbuti/French as example, and (C) the impact of removing CpG sites using San/French and Mbuti/French as example. See caption to S8 Fig for a description of the four phasing methods shown in (A). (PDF) [file pgen.1008552.s009.pdf]

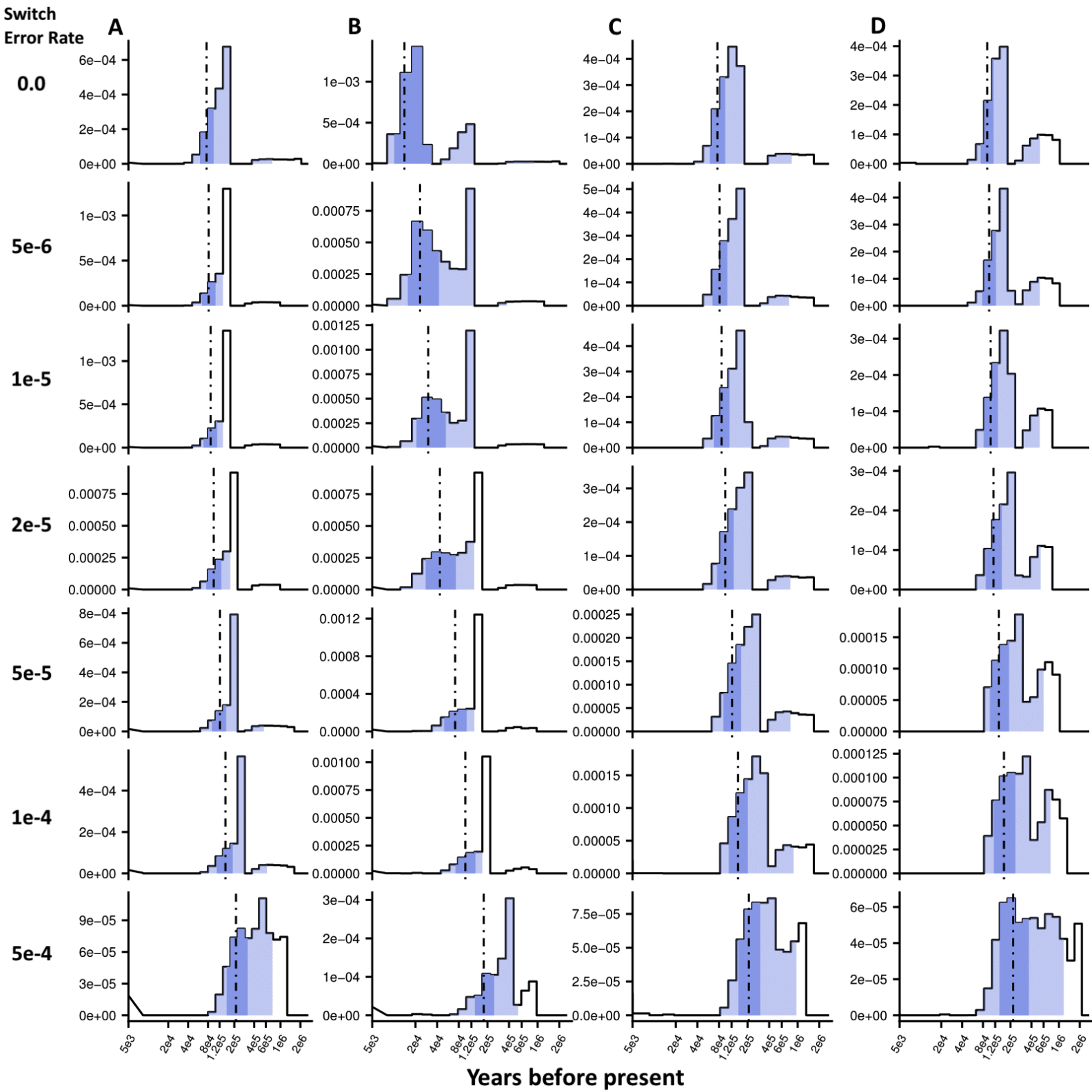

Supplement: S10 Fig — Here we selected the same four simulation scenarios used in S3 Fig, and added phasing switch errors ranging from 5e-6 to 5e-4 per base pair. The overall migration profiles remain relatively consistent for error rates between 5e-6 and 5e-5, with strong effects seen with rates higher than 5e-5, shifting the migration profiles towards older times. (A) Clean split at 75kya. (B) Split at 75kya with symmetric migration between 10-15kya. (C) Split at 75kya with archaic admixture at 5%. (D) Split at 75kya with archaic admixture at 5% and bottleneck in one population. (PDF) [file pgen.1008552.s010.pdf]

**A**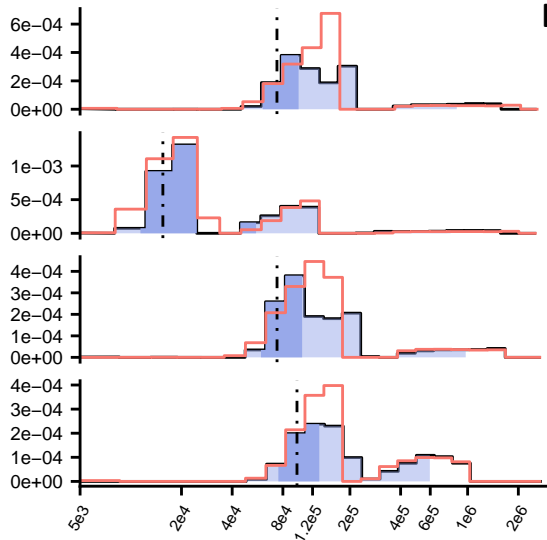**B**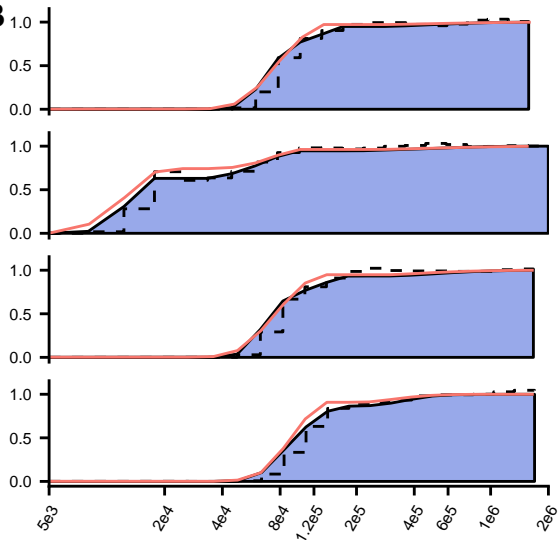

Supplement: S11 Fig — Applying the same four simulation scenarios used in S3 Fig, we here used the genetic map estimated for the human genome (i.e. variable recombination rate across genome) instead of a constant recombination rate. Red lines represent our estimates from using a constant recombination rate 10−8 per generation per bp. (A) Clean split at 75kya. (B) Split at 75kya with symmetric migration between 10-15kya. (C) Split at 75kya with archaic admixture at 5%. (D) Split at 75kya with archaic admixture at 5% and bottleneck in one population. (PDF) [file pgen.1008552.s011.pdf]

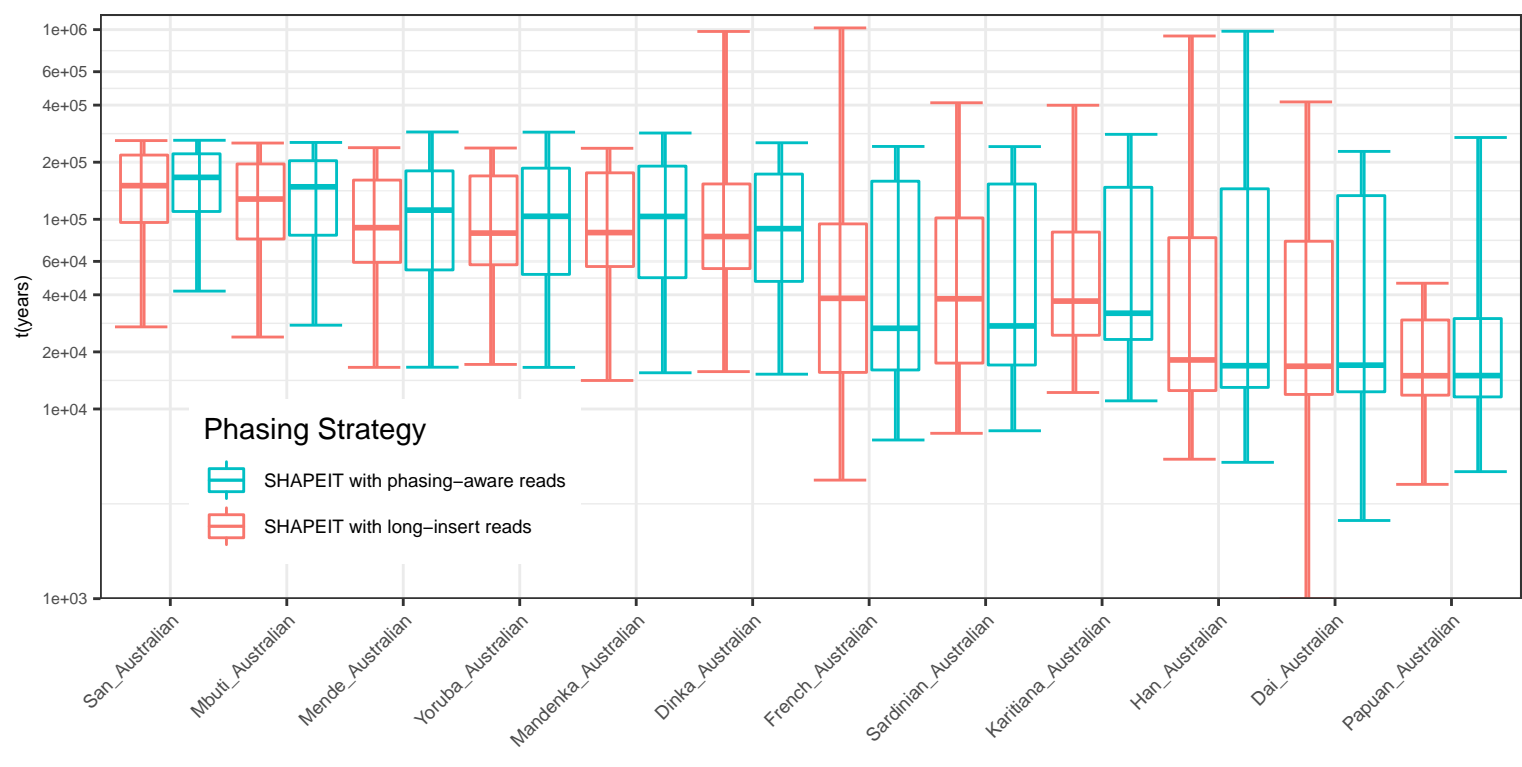

Supplement: S13 Fig — M(t) in quantiles is summarized here between a single Australian and a single individual from worldwide populations. Boxes show the 25% to 75% quantiles of M(t), with bi-directional elongated error bars representing 1% and 99% percentiles. Red color represents the data phased using long-insert reads. Green color represents the standard phased dataset. (PDF) [file pgen.1008552.s013.pdf]

**A**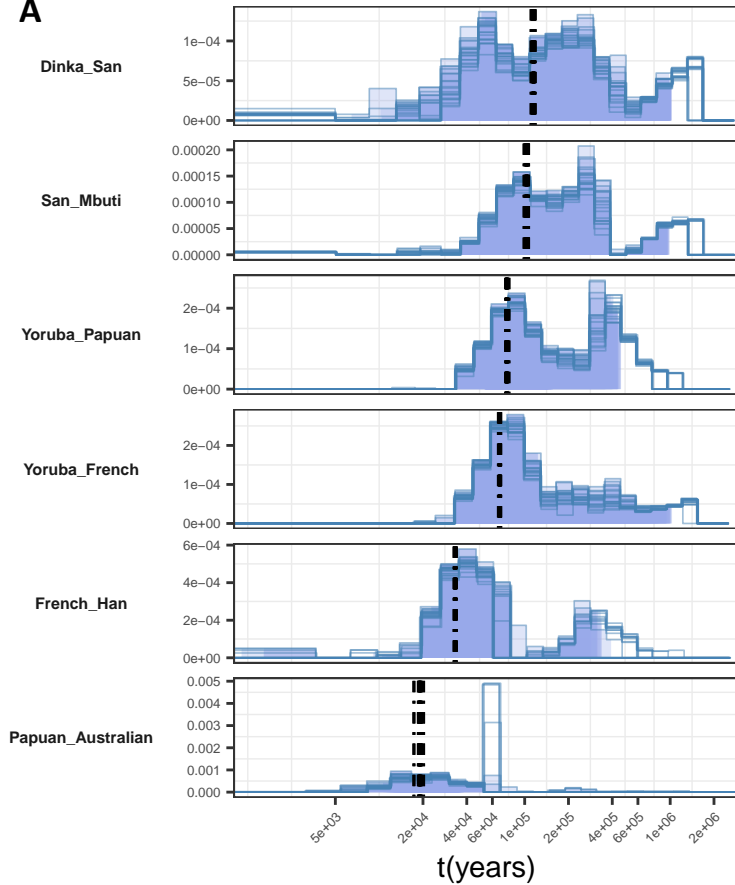**B**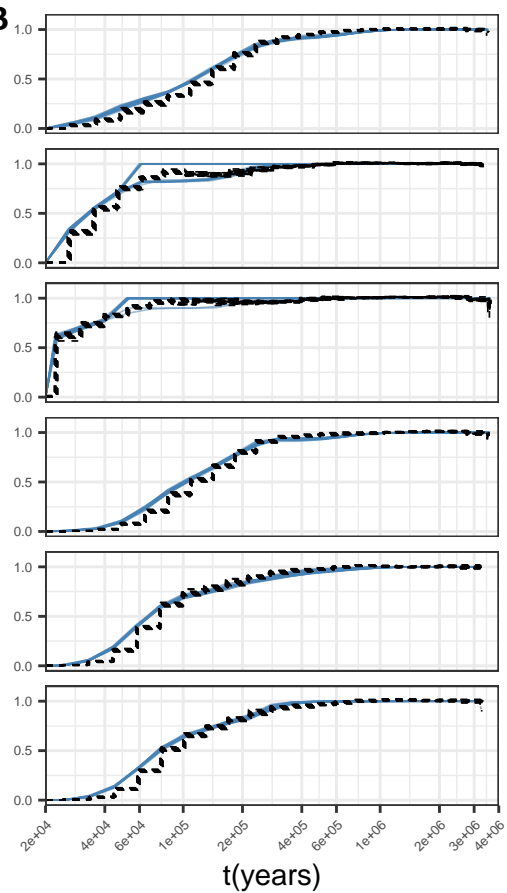

Supplement: S14 Fig — As shown in (A) migration rate m(t) and (B) Cumulative migration probability M(t), the overall inferred profile for each pair is rather consistent across 20 replicates. (PDF) [file pgen.1008552.s014.pdf]
