# Supplementary material for: Tracking human population structure through time from whole genome sequences
Source: PLoS Genet. 2020 Mar 9;16(3):e1008552. doi: 10.1371/journal.pgen.1008552 (PMC7082067; doi:10.1371/journal.pgen.1008552)
Supplement: S3 Fig — The split time T is 75kya in all four cases, and all other parameters are the same as in Fig 2 and as indicated. As shown, the MSMC-IM estimates for N1(t) and N2(t) are close to the inverse coalescence rates, with relatively small effects caused by the migration rate in MSMC-IM which is absent from MSMC2. (PDF) [file pgen.1008552.s003.pdf]

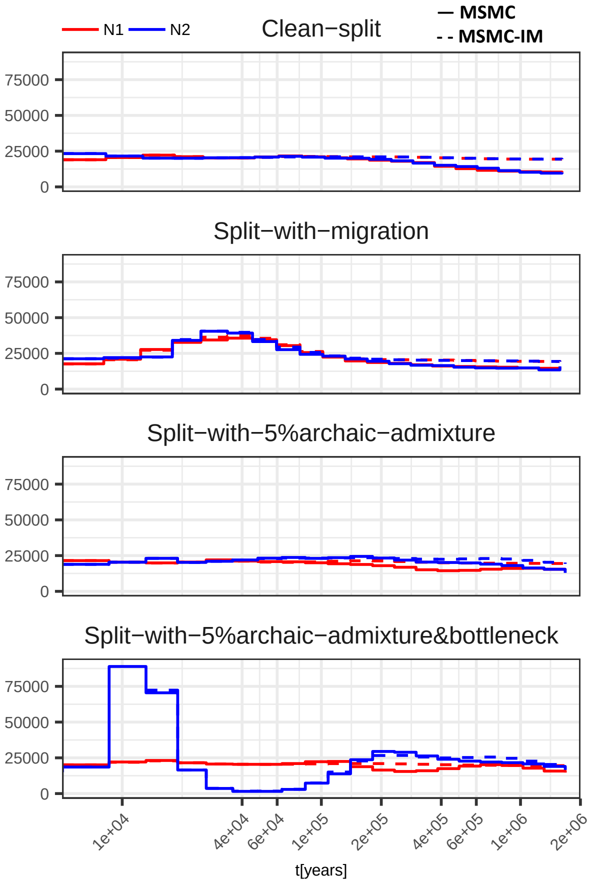

N1
N2
Clean−split
— MSMC
- - MSMC-IM
Split−with−migration
Split−with−5%archaic−admixture
Split−with−5%archaic−admixture&bottleneck
75000
50000
25000
0
1e+04
4e+04
6e+04
1e+05
2e+05
4e+05
6e+05
1e+06
2e+06
t[years]
