# Supplementary material for: Tracking human population structure through time from whole genome sequences
Source: PLoS Genet. 2020 Mar 9;16(3):e1008552. doi: 10.1371/journal.pgen.1008552 (PMC7082067; doi:10.1371/journal.pgen.1008552)
Supplement: S12 Fig — Green lines show the estimates we got from SGDP data for pairs shown on the left (as shown in S4 Fig), which are used as input parameters for the simulation. Red lines show the estimates from applying MSMC-IM on the simulated data. (A) Migration rates m(t). (B) Cumulative migration probabilities M(t) and relative cross-coalescence rates. (PDF) [file pgen.1008552.s012.pdf]

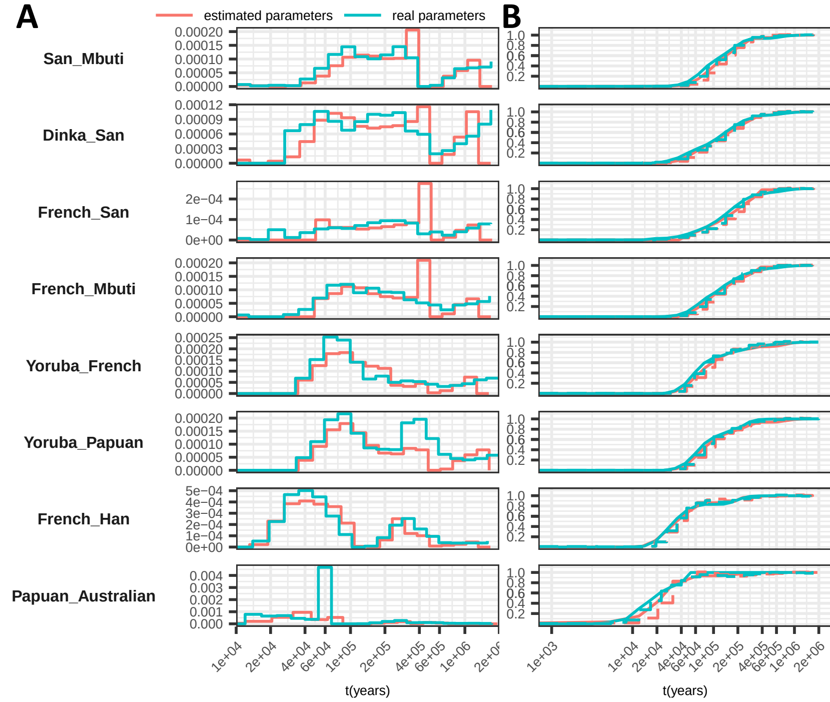
A
B
estimated parameters
real parameters
San_Mbuti
Dinka_San
French_San
French_Mbuti
Yoruba_French
Yoruba_Papuan
French_Han
Papuan_Australian
t(years)
t(years)
